# Peptide‐Ligand Cooperative Interplay Drives Gold Nanoparticle Encapsulation by Protein Cages

**DOI:** 10.1002/smll.73690

**Published:** 2026-05-07

**Authors:** Wenhui Li, Niklas Mucke, Michael Rütten, Tommaso L. Schweers, Tobias Beck, Vikram Jadhao

**Affiliations:** ^1^ Intelligent Systems Engineering Indiana University Bloomington Indiana USA; ^2^ Department of Chemistry Institute of Physical Chemistry University of Hamburg Hamburg Germany; ^3^ Hamburg Centre for Ultrafast Imaging University of Hamburg Hamburg Germany

**Keywords:** encapsulation, encapsulins, gold nanoparticles, molecular dynamics, protein cages

## Abstract

Cargo encapsulation offers broad opportunities in synthetic biology, biocatalysis, and therapeutic delivery, with encapsulins serving as nanoscale reaction chambers or protective carriers. Yet, controlling cargo loading remains challenging. Here, we reveal a molecular‐scale understanding of gold nanoparticle encapsulation in encapsulin protein cages. Experiments investigate how salt concentration, nanoparticle functionalization with ligands, and cargo‐loading peptides influence encapsulation performance. Molecular dynamics simulations connect these experimental observations to the free‐energy landscape governing the initial association of an encapsulin protomer binding to the nanoparticle surface. Simulations reveal three salt‐dependent sets of nanoparticle‐protomer binding free‐energy compared to protomer‐protomer binding energy: much stronger nanoparticle–protomer binding at low salt, slightly stronger nanoparticle–protomer binding in a wide range of intermediate salt, and weakened nanoparticle–protomer attraction at high salt, corresponding to experimental observations of co‐precipitates, nanoparticle encapsulation, and empty cages, respectively. Importantly, the robustness of encapsulation to variations in salt concentration arises from cooperative effects between ligands and peptides: ligands mediate electrostatic attraction and promote peptide extension, while peptides extend the protomer recruitment zone and prevent kinetic trapping. This integrated experimental and computational approach provides molecular‐level insight into encapsulation energetics and peptide‐ligand cooperative interplay, guiding the rational design of bio‐inspired nanocages for selective delivery and templated synthesis.

## Graphical abstract

Gold nanoparticle encapsulation within protein cages is governed by salt‐dependent binding energetics and cooperative peptide–ligand interactions. Ligands enhance electrostatic attraction while peptides extend recruitment and prevent kinetic trapping, enabling robust cargo loading. This molecular‐level insight links experiments and simulations, guiding the rational design of nanocages for controlled encapsulation and targeted delivery.

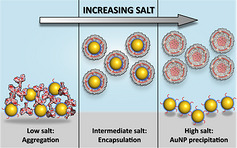

## 1 Introduction

The ability to control assembly at the nanoscale is a fundamental process in living systems and a key strategy for engineering synthetic materials with tailored properties [1, 2, 3, 4]. One prominent theme in nanoscale assembly is compartmentalization: molecular components are spatially confined to specific regions, enabling regulation of biochemical reactions and structural organization [5, 6, 7]. Encapsulation, both in natural and in artificial systems, represents an important example of this principle. Here, molecules, proteins, or nanoparticles are sequestered as cargo within defined structures to enhance stability and/or functionality. Specifically, nanoparticle encapsulation has been employed to improve therapeutic delivery, control catalytic efficiency, and construct novel hybrid materials [8, 9, 10, 11]. A natural model for encapsulation is viral assembly, where capsid proteins spontaneously self‐assemble into protective shells around negatively‐charged nucleic acids through electrostatic interactions [12, 13, 14]. Researchers have extended these electrostatic principles to guide the design of viral capsids, including virus‐like particles (VLPs) that consist solely of the protein shell, for controlled encapsulation of natural and artificial cargo such as enzymes, drugs, polyelectrolytes, quantum dots, and functionalized nanoparticles [15, 16, 17, 18, 19, 20, 21], inspiring the exploration of protein cages as nanocontainers for encapsulation [22, 23, 24] and nanoscale organization [25, 26, 27, 28, 29, 30]. Recent studies have further advanced VLP design by enabling tunable pH‐responsive assembly and controlled encapsulation of protein cargo [31]. Bacterial nanocompartments and microcompartments such as encapsulins and carboxysomes represent a distinct class of naturally evolved protein cages that sequester enzymatic cargo within prokaryotic cells and assemble via entirely protein‐guided and RNA‐independent mechanisms [32, 33, 34, 35, 36, 37, 38]. Bacterial nanocompartments introduce a different encapsulation strategy to recruit cargo, relying on intrinsic protein‐based targeting elements such as cargo loading peptides (CLPs) in encapsulins [39, 40, 41] or scaffold proteins in carboxysomes [42, 43, 44].

Here, we focus on the encapsulin from *Thermotoga maritima* (TmEnc), which is found in bacteria and archaea and naturally functions as a metabolic nanocompartment [33]. TmEnc stands out due to its high thermostability up to 90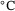 and exclusive T=1 Caspar‐Klug type icosahedral symmetry, in contrast to other encapsulins, which exhibit more complex assembly behaviors including polymorphism [45, 46]. The high stability and rigidity, and defined size monodispersity make TmEnc an ideal nanoscale building block for encapsulation and bioinspired materials design applications [37]. Moreover, encapsulins utilize CLPs that directly bind to the specific recognition sites on the inner surface for highly selective cargo containment. Despite progress in demonstrating that encapsulins can efficiently compartmentalize gold nanoparticles (AuNPs) grafted with positively‐charged ligands and CLPs [28], the molecular‐scale mechanisms driving cargo encapsulation in encapsulin protein cages remain elusive. A fundamental understanding of the link between encapsulation performance, cargo functionalization, and solution conditions is essential for advancing the design of encapsulin‐based nanotechnologies [37] and, more broadly, the development of protein‐coated inorganic nanoparticles for biomedical applications, including drug delivery and photothermal cancer treatments [47, 48]. In our previous study [49], we showed that AuNPs can be encapsulated within TmEnc shells, which in turn can be assembled into ordered binary superlattices through electrostatic interactions, while maintaining the structural integrity of the protein shell. Consistent with these findings, no significant differences were observed in the crystal structures of encapsulin shells before and after AuNP encapsulation [50].

AuNPs, with their well‐established aqueous synthesis and strong optical contrast, in combination with the high thermal stability of the encapsulin shell, represent an excellent hybrid platform for controlled photothermal applications. The ability to adjust the surface charge of protein cages may influence their suitability for future applications as it enables better control over protein corona formation and nanoparticle–cell interactions [51, 52, 53]. Recent studies have shown that AuNPs rapidly acquire a dynamic protein corona in high‐protein‐concentration environments, with the corona composition depending on nanoparticle size and shape [54]. The protein adsorption capacity and structural deformation are governed by surface curvature [55], with the ligand shell dictating the kinetic and thermodynamic evolution of the nanoparticle–protein interface [56]. Thus, encapsulating gold nanoparticles within defined protein cages represents a potential strategy to control corona formation and could guide the development of more stable and functional hybrid nanomaterials in the future.

Using an integrated experimental and computational approach, we investigate the encapsulation of functionalized AuNPs by TmEnc protein subunits (protomers). The co‐assembly of TmEnc protomers and functionalized AuNPs yields three distinct products—AuNP‐protomer co‐precipitates, encapsulated AuNPs, and empty encapsulin cages—in response to increasing salt concentration that progressively screens the electrostatic interactions. The molecular‐scale origins of this co‐assembly behavior are revealed in the analysis of different co‐assembly scenarios produced by altering nanoparticle surface functionalization. Results elucidate how CLP‐mediated interactions complement ligand‐mediated electrostatic and steric forces in encapsulation, and highlight the importance of CLP‐engineered AuNP‐protomer directed interaction and CLP flexibility to encapsulation. The cooperative encapsulation‐promoting effects engineered by peptides and ligands offer generalizable insights applicable to a rational design of bacterial nanocompartments for encapsulation‐based applications [41].

## 2 Results and Discussion

**FIGURE 1:**
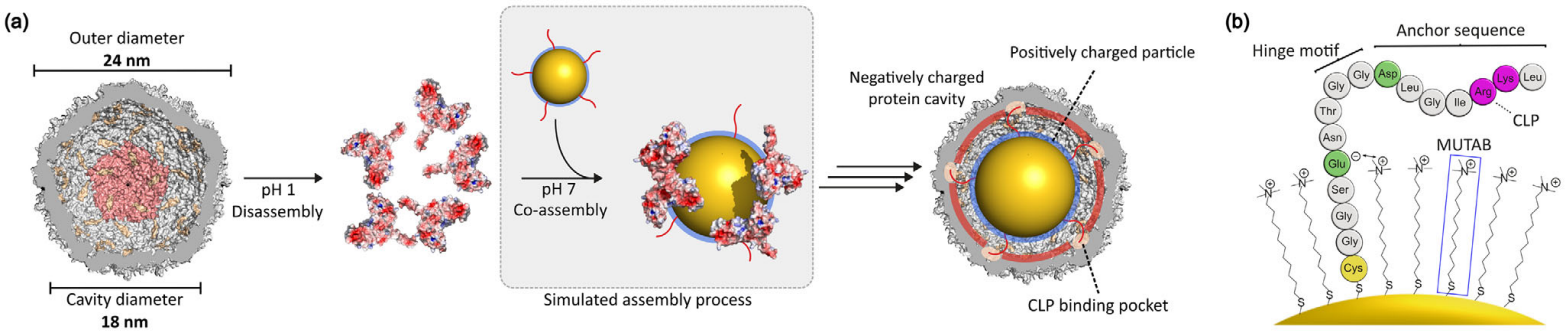
(a) Schematic illustration of the encapsulation process of a functionalized gold nanoparticle (AuNP) within a *Thermotoga maritima* encapsulin protein cage. At pH = 1, the encapsulin disassembles into individual protomers. After return to pH = 7 in the presence of AuNPs coated with positively‐charged ligands and sparsely grafted with cargo‐loading peptides (CLPs), co‐assembly occurs, resulting in a hybrid nanostructure in which the nanoparticle is encapsulated within the protein cavity. The co‐assembly is controlled by the salt‐modulated electrostatic complementarity between the negatively charged protein cavity (red) and the positively charged nanoparticle surface (blue), together with the peptide's specific interaction with the CLP‐binding pockets on the encapsulin protomer surface. (b) Sketch of the surface of a MUTAB‐stabilized AuNP functionalized with a 16‐amino‐acid‐long CLP. The N‐terminal part is buried in the ligand shell, while the C‐terminal anchor sequence binds to the inner encapsulin surface. The schematic amino acids are color‐coded as follows: yellow indicates a sulfur‐containing residue (Cys), grey charge‐neutral residues, green negatively charged residues, and magenta positively charged residues.

TmEnc is a protein cage with inner and outer diameter of ≈ 18 and 24 nm, respectively that assembles from 60 identical protomers (Figure 1a). Each TmEnc protomer has a patchy distribution of partial charges, with a net negative charge of ≈−14e. Cargo encapsulation in TmEnc cages is only possible in vivo during container assembly in the cell. For in vitro encapsulation, TmEnc is first reversibly disassembled in an aqueous solution into protomers at strongly acidic conditions (pH of 1), before adding spherical AuNPs (one AuNP per TmEnc cage) of diameter d≈13.3 nm. Reassembly is initiated by diluting the sample into buffer at pH = 7, together with the functionalized AuNPs, and adjusting the ionic strength by varying the NaCl concentration. (Figure 1a). The AuNP surface is densely grafted with (11‐Mercaptoundecyl)‐*N,N,N*‐trimethylammonium bromide (MUTAB) ligands, which provide high stability over a wide range of pH, ionic strengths, and temperatures. Each ligand molecule carries a charge of +1e on its end atom group. Additionally, the surface is functionalized with ≈9 charge‐neutral peptides for which a truncated 16‐amino acid long CLP from TmEnc is used (Figure 1b). CLPs derived from TmEnc can be divided into three parts: four N‐terminal amino acids containing a cysteine group for covalent binding to the AuNP surface, a middle part comprising a hinge motif with two flexible glycines that facilitates CLP alignment, and a C‐terminal anchor sequence of seven amino acids that exhibits a high biological affinity to the binding pocket on the inner surface of the TmEnc protomer.

The co‐assembly products are analyzed using negative‐stain transmission electron microscopy (TEM), which examines the impact of functionalized ligands and CLPs on the nanoparticle encapsulation performance (see Methods). The AuNPs, protein‐only samples, and co‐assembly products were further characterized by dynamic light scattering (DLS) (Figure S7a), revealing a slight increase in hydrodynamic diameter upon functionalization and co‐assembly (≈16–18 nm for AuNPs; ≈22–23 nm for encapsulin and complexes). Zeta potential measurements (Figure S7b) confirm the positively charged nature of the AuNPs following MUTAB/CLP functionalization (≈+31 to +38 mV). We used thermogravimetric analysis to assess ligand coverage (Figure S8) corresponding to ≈2000 MUTAB ligands per nanoparticle. The stability of the co‐assembly products is evaluated using temperature‐dependent UV–Vis measurements (Figure S9). The protein exhibited no significant spectral changes up to 70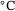, whereas the nanoparticles begin to aggregate and sediment at elevated temperatures. In contrast, the co‐assembly products remained largely stable, with sedimentation occurring only above 60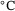. This enhanced stability was further supported by the temperature‐dependent DLS measurements (Figure S10), which showed no significant changes in construct stability up to 60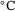, consistent with the higher sensitivity of DLS to particle aggregation.

For molecular dynamics (MD) simulations, we design the models for TmEnc protomer, MUTAB ligand, CLP, AuNP, NaCl salt ions, and water molecules based on the Martini force field [57], which is a widely used coarse‐graining technique in biophysical systems modeling. The Martini coarse‐grained model components in this study are derived from their all‐atom structures which are informed by experiments (Figure S1). MD simulations are used to probe the co‐assembly mechanisms at microscopic scales by extracting the Gibbs free‐energy landscape associated with the protomer–protomer and AuNP‐protomer interactions, and by furnishing the equilibrium structure of the functionalized nanoparticle (see Methods). In the pairwise interaction between a protomer and a functionalized AuNP, the protomer interacts only with a localized region of the AuNP surface. Our simulations are designed to capture this specific contact interaction in order to characterize the initial association step driving the co‐assembly process, and examine the variation of the associated AuNP‐protomer binding energy with salt and AuNP surface functionalization. With this goal in mind and to keep computational costs tractable, the bare AuNP is modeled as a smaller sphere (d≈5 nm) of cross‐sectional area slightly larger than that of a TmEnc protomer. The effects of AuNP size are further examined through MD simulations of a protomer binding to a planar AuNP representative of a very large spherical AuNP (Figure S2).

### 2.1 Co‐Assembly of TmEnc Protomers and Functionalized AuNPs

**FIGURE 2:**
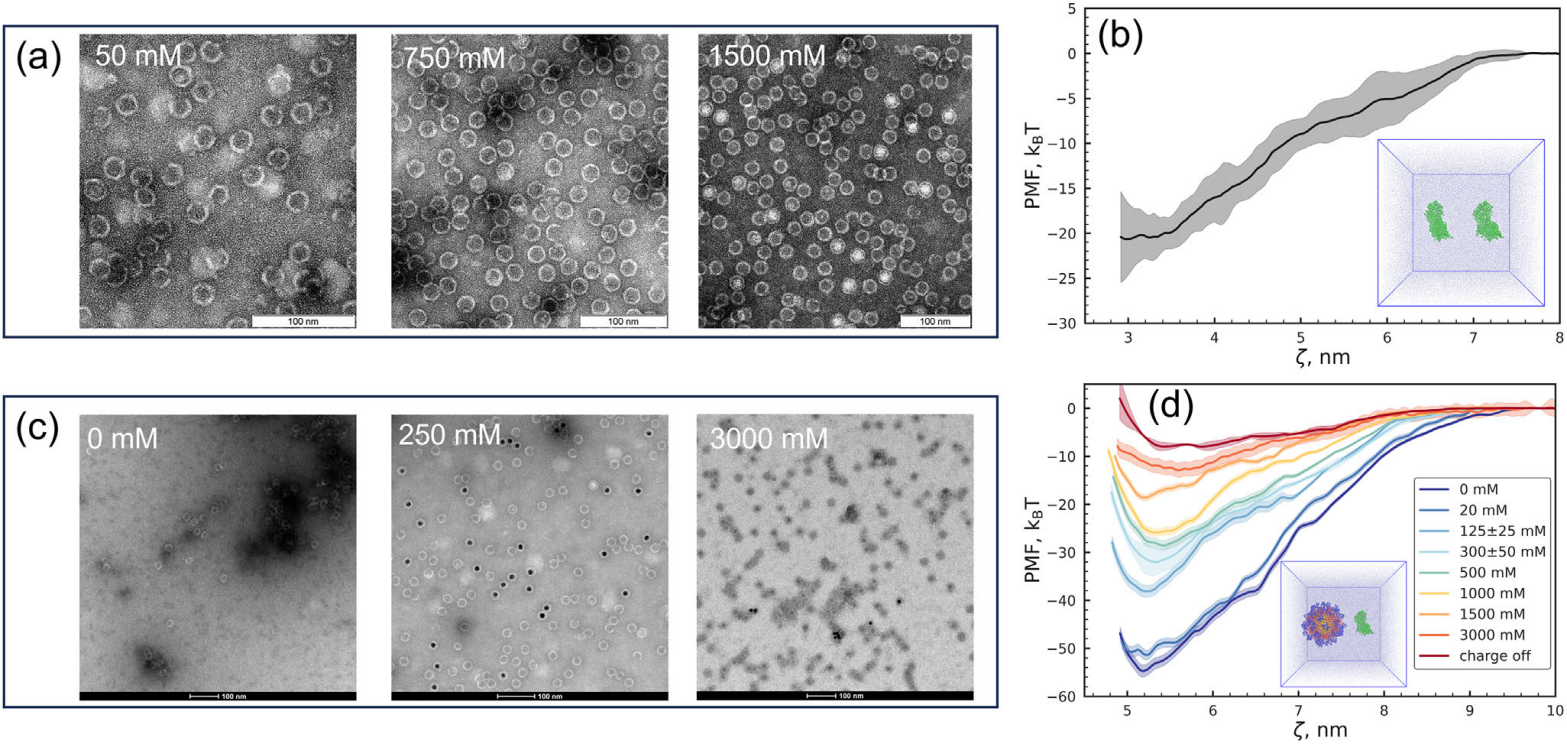
(a) TEM images of the TmEnc‐only assembly at 300 K for a selected set of salt concentrations c indicated on the top left. (b) PMF associated with the interaction between two protomers (inset) obtained from MD simulations averaged over different 0≤c≤1100 mM, as well as for the “charge‐off” case representative of highly screened electrostatic interactions. The gray band represents the standard error indicating the deviations in PMF with changes in c over this wide range. The reaction coordinate ζ represents the center of mass (COM) distance between the two protomers. (c) TEM images of the co‐assembly of encapsulin protomers and AuNPs functionalized with MUTAB ligands and CLPs at the indicated c values on the top left. (d) PMFs associated with the interaction between these functionalized AuNPs and protomers at different c values indicated in the legend. The bands with corresponding lighter colors represent the standard errors. ζ represents the COM distance between the protomer and the AuNP. An example of the simulated systems is shown in the inset.

In experiments at room temperature T=300 K, solutions containing only TmEnc protomers, without the addition of AuNP, show self‐assembly into empty cages for all salt concentrations 0≤c≤3000 mM as evidenced by the TEM images shown in Figure 2a for a selected set of c values. The visualized structure of the encapsulin shell is provided in Figure S3 to identify distinct domains that mediate its self‐assembly and depict the binding interactions between CLP and protomer. Figure 2b shows the protomer‐protomer potential of mean force (PMF) obtained from MD simulations, averaged over different 0≤c≤1100 mM and the “charge‐off” case (Figure S4 shows the individual PMFs). The charges of all particles in the system are set to zero in the “charge off” case, which is representative of highly screened electrostatic interactions. Deviations in PMF due to changes in c are relatively small, indicating that the effective attraction between two protomers is similar regardless of c and characterized by a free‐energy gain ∼−20 $k_{B}T$, which supports the experimental observation of salt‐resistant assembly of TmEnc protomers into cages.

In stark contrast to the TmEnc‐only self‐assembly, salt has a major effect on the co‐assembly of TmEnc protomers and AuNPs functionalized with MUTAB ligands and CLPs (Figure 2c). For low c≲50 mM, co‐precipitates of AuNPs and TmEnc protomers as well as empty TmEnc cages are observed as illustrated with the case of c=0 mM (Figure 2c left) and 50 mM (Figures S5a,b). Encapsulation of AuNPs by TmEnc protomers is observed for 250≤c≤350 mM as illustrated by the TEM images at c=250 mM (Figure 2c middle) and 350 mM (Figure S5c), with approximate encapsulation efficiency of 48% and 55.6%, respectively. We note that while TEM enables a direct visualization of encapsulated versus empty cages, obtaining high‐accuracy estimates for the efficiency metrics based solely on TEM data is affected by the limited sampling statistics and lack of ensemble‐averaged quantification. The encapsulation performance drops at large c, where only empty encapsulin cages are observed. For 1000≲c≲2500 mM, AuNPs are unstable and start to aggregate and sediment out, making encapsulation unfavorable (Figure S5d–g). Unexpectedly, for c≳3000 mM, AuNPs stabilize again, and are found outside the empty cages or in smaller aggregates as illustrated by the TEM image at c=3000 mM (Figure 2c right).

The PMFs associated with the AuNP‐protomer interaction show that, unlike the protomer–protomer free‐energy drive, the free‐energy F characterizing the co‐assembly process is sensitive to large variations in c (Figure 2d). The PMF curves can be classified into three distinct sets based on the free‐energy minimum $F_{m}$, characterizing the strength of the AuNP‐protomer bond, and the long‐range decay of F signifying the protomer recruitment potential of AuNP. For very low c (≲20 mM), F exhibits a deep minimum ($F_{m}≲-50$ $k_{B}T$) and a gradual long‐range decay (F≈0 for ζ⪆9 nm). This strong and long‐range AuNP‐protomer attraction causes rapid recruitment of protomers by AuNPs, leading to kinetic trapping and the formation of AuNP‐protomer co‐precipitates. The co‐precipitation depletes all AuNPs and some of the proteins from the solution, leaving the remaining protomers to self‐assemble into empty TmEnc cages. Another set of PMF curves is associated with very high c (≳3000 mM) and the “charge‐off” case. Here, F exhibits a shallow minimum ($F_{m}>-20$ $k_{B}T$) and a rapid long‐range decay (F≈0 for ζ⪆8 nm), signaling a shrinking of the protomer recruitment zone. This weaker AuNP‐protomer attraction compared to the protomer–protomer attraction (Figure 2b) makes encapsulation free‐energetically unfavorable, explaining the observation of AuNPs outside TmEnc cages at c=3000 mM.

**FIGURE 3:**
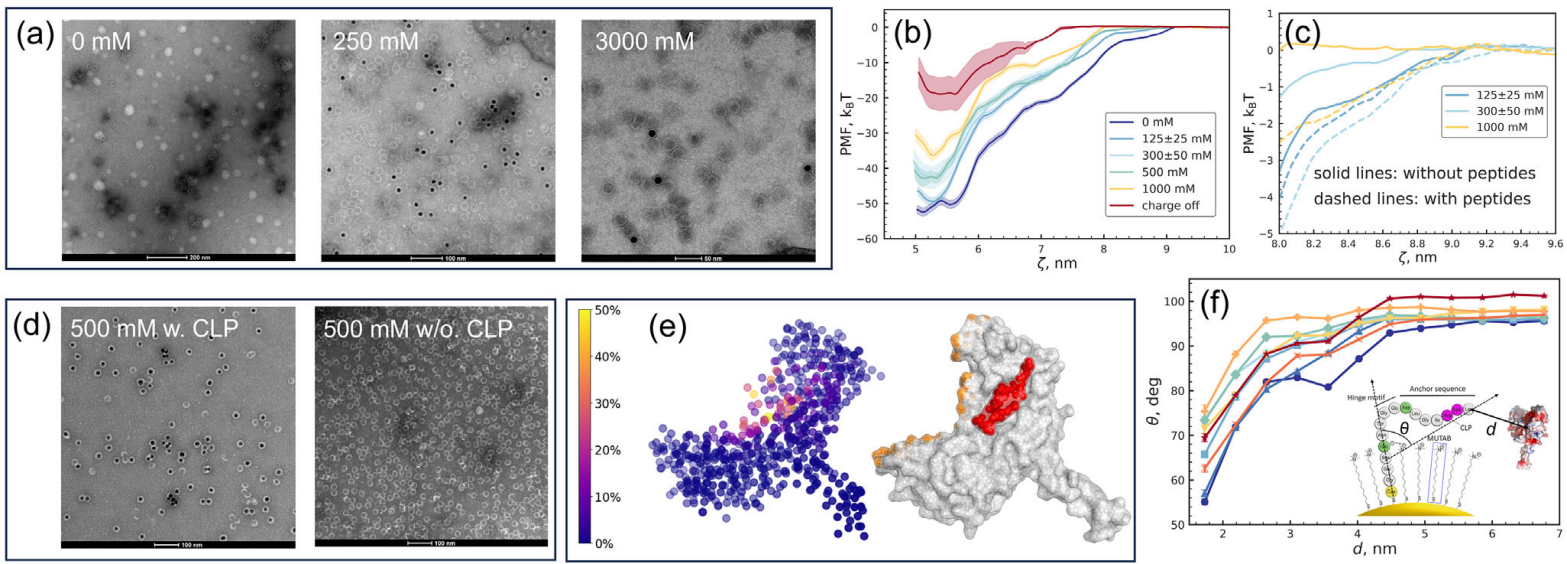
(a) TEM images of the co‐assembly of TmEnc protomers and AuNPs functionalized only with MUTAB ligands at the indicated salt concentration c. (b) PMF between these functionalized AuNPs and protomers at different c; the bands with corresponding lighter colors represent the standard errors. (c) The comparison of the PMFs at c=125±25,300±50,1000 mM for 8≤ζ≤9.6 nm with the PMFs (dashed lines) from Figure 2d for the case where AuNP is functionalized with both MUTAB ligands and CLPs. (d) TEM images of (left) the co‐assembly of TmEnc protomers with AuNPs functionalized with MUTAB and CLP at 500 mM salt, and (right) the empty, self‐assembled TmEnc cages formed in the presence of AuNPs functionalized only with MUTAB ligands at the same salt concentration. (e) The surface of a TmEnc protomer color coded (left image) based on probability of finding the protomer within a distance of 0.5 nm from the CLP anchor sequence beads when the separation between MUTAB and CLP‐functionalized AuNP and protomer is in the range of 5 to 5.5 nm. The right image of a TmEnc protomer shows the peptide binding pocket (red region) and the protomer‐protomer binding site (orange region). (f) The angle θ formed by the vector of the CLP backbone (N‐Term_surface_‐CGGSENTGGDLGIRKL‐C‐Term_protein_) beads of the first and fourth amino acids (which is part of the N‐terminal of the CLP) and the vector of the CLP backbone beads of the fourth and the last amino acids versus the distance d (inset) between the backbone bead of the last amino acid and the protomer COM. Different colors correspond to the legend in Figure 2d. The inset also shows the CLP composition and its anchor sequence in red, along with the MUTAB ligands.

The final set of PMF curves are identified by intermediate c values (125±25≲c≲1000 mM) for which the free‐energies are clearly separated from the free‐energies associated with the aforementioned regimes. F is characterized with an attraction ($-25≲F_{m}≲-50$ $k_{B}T$) stronger than the protomer–protomer attraction and yet weak enough to circumvent kinetic trapping. Further, F exhibits a relatively slow long‐range decay (F≈0 for ζ⪆9 nm), creating an expanded protomer recruitment zone. The experimentally observed enabling zone for AuNP encapsulation can be linked to the free‐energy landscape associated with c=300±50 mM, wherein the PMF curves exhibit minimal variation and are characterized with an AuNP‐protomer binding energy $|F_{m}|≳30$ $k_{B}T$. $|F_{m}|$ decreases with increasing c up to 1000 mM, however, the AuNP‐protomer attraction continues to be strong compared to the protomer–protomer attraction and the long‐range behavior of F remains conducive for encapsulation. Encapsulation is thus forecasted, if AuNPs could be stabilized, for 300≲c≲1000 mM. This prediction is borne out as robust encapsulation is observed at 500 mM (Figure 3d).

Experimental systems using larger AuNPs of diameter 13.3 nm are expected to be associated with a stronger AuNP‐protomer attraction compared to the attraction predicted by simulations in Figure 2d due to slightly greater AuNP‐protomer contact area arising from the reduced curvature of the larger particles, which enhances van der Waals attraction. For example, we expect the attraction for the high c cases to be slightly stronger in experiments. An estimate for a lower bound of $F_{m}$ can obtained via simulations of protomers near a gold plane (representing a very large AuNP) and reveals that systems with highly screened electrostatic interactions continue to exhibit $F_{m}≳-20$ $k_{B}T$. A similar analysis establishes a lower bound of the free‐energy gain for successful encapsulation to be $F_{m}\approx -50$ $k_{B}T$ (Figure S2).

### 2.2 Peptide‐Mediated Mechanisms Driving Encapsulation

To decouple the roles of CLPs and ligands in setting up the AuNP‐protomer attraction drive shown in Figure 2d, we repeat co‐assembly studies for AuNPs functionalized only with MUTAB ligands, finding a similar broad co‐assembly behavior (Figure 3a,b). Co‐precipitation is observed at very low c, and is linked to a strong AuNP‐protomer attraction, and AuNPs are found outside the empty encapsulin cages at very high c, which is attributed to the relatively weak AuNP‐protomer attraction as illustrated by the “charge‐off” case in Figure 3b. AuNP encapsulation is observed without CLPs for a narrow range of c, as shown in Figure 3a at c=250 mM with encapsulation efficiency of 20%.

The PMF curves in Figure 3b for 300±50 mM signal that the free‐energetic drive associated with encapsulation is set up by the attractive MUTAB‐TmEnc electrostatic force, and does not require CLPs. A closer examination sheds light on two peptide‐specific mechanisms affecting the energy landscape. The long‐range (ζ≥8 nm) AuNP‐protomer attraction is weaker without CLPs (Figure 3c) and decreases significantly as c is increased from 125±25 mM to 1000 mM. In contrast, the electrostatic screening effects on the long‐range AuNP‐protomer attraction are insignificant for AuNPs functionalized with CLPs over the same salt range. Thus, CLPs protect the protomer recruitment zone conducive for encapsulation against changes in ionic strength, which can be attributed to the CLP‐TmEnc attraction. These simulation‐derived insights are validated in experiments that encapsulation occurs at 500 mM for AuNPs functionalized with both MUTAB and CLP, whereas only empty TmEnc cages form when the AuNPs are functionalized with MUTAB alone (Figure 3d).

A comparison of Figures 2d and 3b also shows that the short‐range (ζ≲6 nm) AuNP‐protomer attraction is stronger without CLPs, which can be attributed to the absence of steric hindrance offered by CLPs to protomers that come too close to AuNP. For example, encapsulation without CLPs is associated with a higher AuNP‐protomer binding energy ($|F_{m}|\approx 42$ $k_{B}T$) compared to AuNP encapsulation with CLPs at the same c ($|F_{m}|\approx 30$ $k_{B}T$). Modest reduction in c can significantly enhance this short‐range attraction, which increases the energy barrier for protomers to rearrange, making it easier for AuNPs and protomers to get kinetically trapped and co‐precipitate. Thus, overall, the CLPs enable encapsulation over a wider range of ionic strength by offering stronger long‐range attraction that enhances protomer recruitment and by producing short‐range steric hindrance that inhibits protomer kinetic trapping.

We identify the CLP binding sites on the TmEnc protomer surface by computing the probability of finding a CLP anchor sequence bead within 0.5 nm of a protomer bead for AuNP‐protomer COM separation between 5 and 5.5 nm, where the AuNP‐protomer free‐energy is near its minimum (see Supporting Information). This binding probability changes minimally over a wide range of c, and is computed as an average utilizing the simulation trajectory data generated for cases shown in Figure 2d. The parts of the protomer surface with high binding probability (left image in Figure 3e) correlate well with the CLP‐binding pocket (red) and the region (orange) corresponding to the protomer–protomer binding site (right image in Figure 3e, known from the literature). The close proximity of CLPs to the protomer‐docking region enhances the probability of the AuNP‐protomer complex recruiting another protomer via the CLP‐protomer attraction. Thus, CLPs engineer a “directed force” between AuNP and TmEnc protomer that secures AuNP‐protomer binding and enhances subsequent protomer docking, facilitating encapsulation.

The four N‐terminal amino acids of a CLP backbone are confined by the ligand shell and exhibit limited movement. The remaining amino acids have more freedom to rotate and extend. The angle θ (Figure 3d inset) formed by the distance vector between the CLP backbone beads of the first (bonded to AuNP) and fourth amino acids and the distance vector between the CLP backbone beads of the fourth and last amino acids is extracted to assess CLP's response to incoming protomers at different c shown in Figure 2d using the corresponding simulation trajectory data (see Supporting Information). When the protomer is far from the CLPs, as quantified by the distance d≳4 nm between the last CLP backbone bead and the protomer COM, θ assumes a constant average value $\approx 97\pm 3^{\circ }$ (Figure 3f), characteristic of a functionalized AuNP conformation where all CLPs adhere the ligand shell. However, as the protomer approaches closer to the CLPs, indicated by a decreasing d, θ decreases sharply, signaling a CLP rotation away from the ligand shell and toward the protomer. This rotational response shows that CLPs are highly flexible and act as “protomer sensors” to enable effective protomer capture, facilitating encapsulation.

### 2.3 Ligand‐Mediated Mechanisms Underlying Encapsulation

**FIGURE 4:**
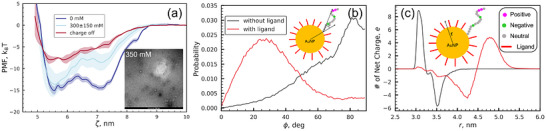
(a) PMFs associated with the AuNP‐protomer interaction for AuNP functionalized with CLPs and negatively charged MUA ligands at different salt concentrations c indicated in the legend and for the “charge‐off” case. The bands with corresponding lighter colors represent the standard errors. Inset is the TEM image of the co‐assembly of TmEnc protomers and AuNPs functionalized with MUA ligands and CLPs at c=350 mM. (b) Probability that a CLP orients at an angle ϕ for AuNPs grafted without (black line) and with (red line) MUTAB ligands, where ϕ is the angle between the distance vector extending from the AuNP center to the CLP backbone bead bonded to AuNP (the first bead) and the distance vector along the CLP backbone from the first to the fourth bead. The inset shows a schematic of the AuNP (large circle) grafted with CLP (small circles) and ligands (short red lines), indicating the angle ϕ. (c) The number of net charges of peptide versus the radial coordinate r for AuNP grafted without (black line) and with (red line) ligands. The amino acids constituting the CLP are represented by different colors based on their charge as shown in the legend.

Figures 2d and 3b highlight that the electrostatic attraction between the positively‐charged MUTAB ligands and TmEnc protomers (bearing a net negative charge) is critical to generate the AuNP‐protomer attraction that promotes encapsulation. Switching to AuNPs grafted with CLPs and negatively‐charged mercaptoundecanoic acid (MUA) ligands produces an entirely different free‐energy landscape (Figure 4a). While the “charge‐off” case is predictably identical in Figures 2d and 4a, decreasing c down to the no salt limit yields a change of only ≈5 $k_{B}T$ (from −10 to −15 $k_{B}T$) in the AuNP‐protomer binding free‐energy for MUA‐functionalized AuNPs. In stark contrast, over ≈45 $k_{B}T$ change (from −10 to −55 $k_{B}T$; Figure 2d) is observed for the MUTAB‐functionalized AuNPs for the same salt range, which covers the zone where this binding energy noticeably exceeds the protomer–protomer binding energy. Moreover, the electrostatic repulsion between the MUA ligands and the TmEnc protomers produces a net AuNP‐protomer repulsion for 6≲ζ≲7 nm. Encapsulation of AuNPs grafted with negatively‐charged ligands is thus free‐energetically unfavorable regardless of salt, a prediction borne out in experiments that find TmEnc cages with no encapsulated AuNPs, as illustrated by the TEM image at 350 mM in Figure 4a inset.

Encapsulation‐promoting effects related to CLP flexibility and CLP‐engineered AuNP‐protomer directed interaction can only manifest if CLPs are suitably oriented relative to the AuNP surface. For example, CLPs extending outward from the AuNP center and orienting perpendicular to the AuNP surface have a higher chance of recruiting nearby protomers compared to CLPs adhering (orienting parallel to) the AuNP surface. Entropy associated with the amino acid chain favors CLPs extending outward, while the hydrophobic forces favor the CLPs adhering the AuNP surface to minimize contact between the solvent molecules and CLPs/AuNP.

MD simulations of an AuNP functionalized with 50 CLPs in the presence and absence of MUTAB ligands at salt concentrations c=0,200,500,800 mM are performed to determine the equilibrium CLP orientation, measured in terms of the angle ϕ (Figure 4b inset) between the distance vector extending from the AuNP center to the first CLP backbone bead and the distance vector along the CLP backbone from the first to the fourth bead. CLPs oriented perpendicular to the AuNP surface have smaller $\phi ∼0^{\circ }$ values while CLPs adhering the AuNP surface exhibit $\phi ∼90^{\circ }$.

Figure 4b shows the probability distribution p(ϕ) for a CLP to have an orientation $0^{\circ }\leq \phi \leq 90^{\circ }$ computed by averaging the data for all CLPs and c values. p(ϕ) changes minimally for different c. For AuNP functionalized only with CLPs, p(ϕ) is large for $\phi >60^{\circ }$ and peaks at $\approx 80^{\circ }$, indicating that the CLPs prefer to orient parallel to the AuNP surface. In stark contrast, for AuNP functionalized with both ligands and CLPs, p(ϕ) is very small for $\phi >60^{\circ }$ and peaks at $\approx 25^{\circ }$, indicating that the CLPs tilt significantly away from the AuNP surface. This change can be attributed to the ligand‐CLP steric repulsion which amplifies the entropic effects favoring the CLPs to extend outward and orient closer to the perpendicular direction relative to the AuNP surface.

While the CLP is overall electroneutral, it has two positively‐charged beads as part of the anchor sequence (head), which interact with the patches of charges on the TmEnc protomer surface, and two negatively charged beads in the middle (Figure 4c inset). By affecting the CLP orientation, ligands also alter the exposure of these charged amino acid residues, measured as the number of net charges associated with a functionalized AuNP at different radial coordinate r (Figure 4c). In the absence of grafted ligands, a prominent positive charge peak just outside the bare AuNP at r≈3 nm is followed by a negative charge peak at r≈3.5 nm, indicating that the positively‐charged CLP head beads are closer to the AuNP surface likely due to the dominance of the hydrophobic forces. On the other hand, for AuNPs grafted with both ligands and CLPs, the peaks occur farther from the AuNP surface and in reverse order with the negative charge peak at r≈4.3 nm followed by a positive charge peak at r≈4.8 nm, indicating that CLPs are extended outward from the AuNP surface. This dramatic change in the structural conformation of the functionalized AuNP attributed to the ligand‐CLP steric repulsion and the associated enhancement of entropic effects, makes AuNPs more likely to sense and respond to incoming protomers, promoting encapsulation.

### 2.4 Effects of Temperature

**FIGURE 5:**
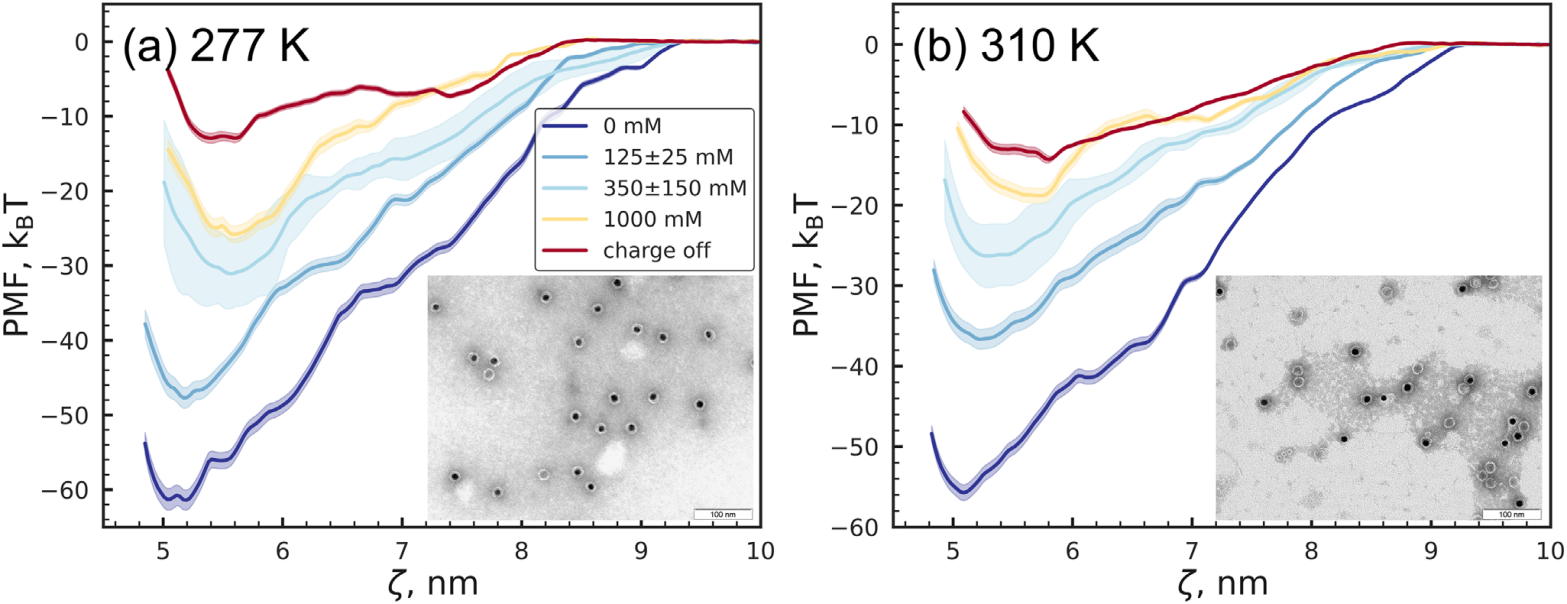
PMFs associated with the AuNP‐protomer interaction for AuNPs functionalized with CLPs and MUTAB ligands at temperature 277 K (a) and 310 K (b) for salt concentrations indicated in the legend in (a). The bands with corresponding lighter colors represent the standard errors. The insets show the TEM images of the co‐assembly of AuNPs and TmEnc protomers at 350 mM.

The cooperative interplay of CLPs and MUTAB ligands also protects AuNP encapsulation by TmEnc cages under temperature variations as evidenced by the free‐energy landscapes extracted at 277 and 310 K (Figure 5): Encapsulation is expected to be feasible in a similar zone of 350±150 mM as observed for 300 K based on the relatively gradual long‐range decay of F and a suitable AuNP‐protomer binding free energy $|F_{m}|≳25$ $k_{B}T$. This prediction is borne out in experiments which observe encapsulated AuNPs at c=350 mM (Figure 5a,b inset). The overall free‐energy F associated with the AuNP‐protomer interaction at 277 and 310 K also signals that the very low (e.g., ∼0 mM) salt and very high salt (“charge‐off”) conditions will continue to favor co‐precipitation and no AuNP encapsulation respectively. The robustness of encapsulation of MUTAB and CLP‐functionalized AuNPs across a wide range of salt and temperature demonstrates the broad application scope of using encapsulin‐based systems.

## 3 Conclusions

Protein self‐assembly around cargo does not follow a universal mechanism; it depends on the nature of the cargo and how the cargo interacts with the protomers. While the protomers self‐assemble into cages through protomer‐protomer attraction, the inner surface of each protomer should favorably interact with the cargo to ensure efficient encapsulation. In the case of VLPs, encapsulation is facilitated through electrostatic interactions, inspiring the functionalization of cargo with negatively‐charged ligands to enable a favorable interaction with the positively‐charged inner surface of VLPs. For example, capsid proteins derived from plant viruses such as brome mosaic virus and cowpea chlorotic mottle virus were used to encapsulate AuNPs grafted with negatively‐charged ligands, revealing a critical threshold of nanoparticle surface charge density for encapsulation [58, 59, 60]. Theoretical studies have advanced the assembly engineering of VLP proteins around functionalized nanoparticles [61, 62, 63].

While both VLPs and bacterial nanocompartments offer advantages such as biocompatibility, structural precision, and tunable cargo loading, their distinct assembly principles and encapsulation mechanisms enable different applications in nanotechnology, catalysis, and targeted delivery [10, 19, 32, 41]. During cargo encapsulation by carboxysomes, co‐assembly of proteins, and cargo occurs in the presence of scaffold proteins [42, 43], whose properties (e.g., length, cargo affinity) determine encapsulation performance, as elucidated in recent computational studies [64, 65]. Encapsulins have negatively‐charged inner surfaces and utilize CLPs for cargo loading [39, 40]. These unique features inspire a different cargo functionalization strategy e.g., grafting the surface with positively‐charged ligands and CLPs. Here, we have elucidated a molecular‐scale mechanistic understanding of the link between encapsulation performance, cargo functionalization, and solution conditions for encapsulin‐based systems.

We have shown that in the co‐assembly of TmEnc protomers and functionalized AuNPs, the strength and long‐range behavior of the effective AuNP‐protomer interaction determine whether the encapsulin‐cargo system produces encapsulation as a favorable co‐assembly product. Encapsulation of AuNPs functionalized with MUTAB ligands and CLPs is forecasted at ambient pressure and 300 K over a broad range of salt 125≲c≲1000 mM, assuming AuNPs can be stabilized over this range, and is observed experimentally from 250 to 500 mM. Encapsulation is characterized with a AuNP‐protomer binding free‐energy of ≳25 $k_{B}T$, and is protected under temperature variations to 277 and 310 K. Under these solution and thermodynamic conditions, the AuNP‐protomer attraction is in the “Goldilocks zone”—stronger than the protomer–protomer attraction but weak enough to circumvent AuNP‐protomer kinetic trapping. Encapsulation is not observed at high ionic strengths where the AuNP‐protomer attraction decays rapidly and is too weak to overcome the protomer‐protomer binding energy of ≈20 $k_{B}T$ to form empty cages. On the other hand, the AuNP‐protomer attraction becomes too strong and long‐ranged at very low ionic strengths, leading to kinetically trapped AuNP‐protomer co‐precipitates.

We have demonstrated that the peptide‐ligand cooperative interplay drives cargo encapsulation in protein cages, thus expanding the design space for functional nanocompartments. Ligands produce the electrostatic nanoparticle‐protomer attraction that sets up the free‐energetic drive to encapsulate and amplify the entropic effects favoring the CLPs to extend outward. CLPs counteract electrostatic screening, extending nanoparticle's protomer recruitment zone, and produce short‐range steric hindrance, inhibiting protomer kinetic trapping. Further, we show that CLP‐engineered AuNP‐protomer “directed” force and CLP flexibility are critical to encapsulation—the former secures AuNP‐protomer binding and enhances protomer–protomer binding, while the latter ensures effective protomer sensing and capture.

Our current understanding of the primary role of ligands in encapsulin‐based sequestering of cargo is that ligands are needed to stabilize the cargo, e.g., to prevent it from precipitating out, which would indicate that they are only incidental to encapsulation. We demonstrate that appropriately‐charged ligands are in fact indispensable to cargo encapsulation by encapsulin protomers. For example, AuNP stability can be achieved by using negatively‐charged ligands (MUA), however, AuNPs functionalized with these ligands (and CLPs) are not encapsulated at any salt concentration. Moreover, ligands are critical to the interplay between the competing entropic and hydrophobic forces that produce the equilibrium CLP orientation conducive for encapsulation.

A few limitations of our computational approach need to be noted. The standard Martini model does not permit an explicit representation of inorganic (metal) atoms. As a result, the model AuNP is made up of organic beads [57], which can exhibit a mild attraction toward the protomer beads. We expect this attraction to have a minimal effect on the AuNP‐protomer binding energetics due to the densely grafted layer of ligands, which substantially screens these beads from getting exposed to the protomer. The complete encapsulation co‐assembly pathway and associated timescales involve interactions between protomer‐coated AuNPs and dispersed protomers, which were not explored here but can have important implications. We expect our findings to guide these studies, e.g., via the PMFs produced here that can inform the design of coarser‐grained models [66] capable of probing the entire cargo‐protomer co‐assembly pathway; these developments are underway.

While the encapsulation strategy has been successfully demonstrated, some experimental limitations remain. The current assays primarily rely on endpoint measurements, limiting insight into the real‐time dynamics of cage assembly and cargo loading. This constrains our understanding of transient intermediates and kinetic bottlenecks, which may be critical for optimizing encapsulation efficiency. Time‐resolved studies, such as small‐angle X‐ray scattering (SAXS), could offer deeper insight into the time‐dependent steps of the encapsulation process [67]. Additionally, since loading efficiency is currently inferred from bulk measurements, single‐particle techniques may be required to resolve heterogeneity across individual cages.

The two features of the functionalized AuNP cargo–the positive surface charge provided by MUTAB ligands and the specific recognition enabled by charge‐neutral peptide binding with the encapsulin interior–act together to promote desirable co‐assembly products and suppress undesired aggregation pathways. This cooperative effect thus describes a functional synergy between electrostatic attraction and sequence‐specific recognition rather than allosteric modulation of binding affinity, known in enzymes. The peptide–ligand cooperative interplay is unique to the AuNP‐encapsulin assembly and fundamentally differs from the purely charge‐driven encapsulation mechanisms [19]. Such a combined interaction mechanism reveals a relatively under‐explored pathway for recruiting cargo into encapsulin cages.

The broader potential of peptide‐ligand cooperative interplay in nanoparticle encapsulation opens promising directions. The modularity of the system could be further leveraged to encapsulate a wide variety of cargos, enabling applications in catalysis, imaging, and drug delivery. Moreover, the ability to form highly ordered nanoparticle lattices using protein cages as scaffolds may pave the way for bioinspired metamaterials with tunable optical or electronic properties [68]. Future work may also explore dynamic or stimuli‐responsive versions of the system, in which encapsulation is reversible or regulated by environmental triggers–extending the utility of protein cages as programmable nanoscale containers.

## 4 Methods

### 4.1 Experimental Methods

For small‐scale encapsulation experiments, 7.5 μg of encapsulin (2 μL of a 3.75 mg mL^−1^ stock) was mixed with 8 μL of 10 mM phosphate buffer (pH 1.0, 0 M NaCl) and incubated at 4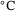 for 1 h. The disassembled protein was then added to 1.4 mL of reassembly buffer (20 mM phosphate, pH 7.0) supplemented with different NaCl concentrations. To each reassembly reaction, 15 μL of MUTAB‐ and CLP‐functionalized AuNPs (1.76 nmol mL^−1^) were added, resulting in a 1:1 protein‐to‐AuNP molar ratio. AuNPs are further characterized by dynamic light scattering (DLS) (Figure S7a), ζ‐potential measurements (Figure S7b), and thermogravimetric analysis (TGA) (Figure S8) to assess their hydrodynamic size, surface charge, and ligand coverage, respectively. The samples were incubated overnight at 4, 27, or 37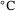. After incubation, samples were centrifuged at 500 × *g* for 1 min to remove aggregates and precipitates. The supernatant was concentrated to 50 μL using centrifugal filtration (Amicon Ultra‐15 Centrifugal Filters MWCO 30 kDa) and stored at 4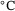. The co‐assembly constructs are also analyzed by DLS, demonstrating that no significant changes in hydrodynamic size occur upon encapsulation (Figure S10).

TEM analysis was performed using carbon‐coated copper grids (400 mesh, Ted Pella, 01814‐F‐X) using a JEOL JEM‐1011 and a FEI Tecnai G2 Spirit Twin, both operated at 100 kV acceleration voltage. For negatively stained samples, a 2% (w/v) aqueous uranyl acetate solution was used, filtered through a 0.22 μm syringe filter prior to use. Grids were floated on 10 μL sample droplets placed on parafilm and incubated for 1 min, followed by three washes with ultrapure water. Subsequently, grids were stained by a 60 s incubation on a 2% uranyl acetate droplet, blotted, and air‐dried.

Additional details on the pH‐dependent disassembly and reassembly, nanoparticle synthesis and surface modification, as well as protein production and purification, and characterization techniques (DLS, ζ‐potential, TGA, and temperature‐dependent UV–Vis) are provided in the Supporting Information.

### 4.2 Simulation Methods

The TmEnc protomer, MUTAB and MUA ligands, CLP, AuNP, NaCl salt ions, and water molecules were modeled using the Martini force field (version 3.0) [57] which maps 2, 3, or 4 heavy atoms into one bead of tiny, small, or regular size respectively. With this coarse‐graining, the CLP has a backbone with multiple side chains and an end‐to‐end distance of ≈1.9 nm, while the ligand is a linear chain of beads with an end‐to‐end distance of ≈1.2 nm. Non‐bonded interactions between beads included pairwise van der Waals interactions, represented by a 12‐6 Lennard‐Jones potential, and Coulomb interactions. Bonded interactions consisted of bond stretching and angle bending, both described by harmonic potentials. Water was modeled using a single “tiny” bead representation. The PMF trends from this non‐polarizable model were qualitatively similar to the results obtained with the Martini 2.2 force field with polarizable water model (Figure S7). Ligands and CLPs were randomly grafted onto the AuNP surface by connecting their bonding atoms to the nearest unique gold atoms via a harmonic potential. AuNP was functionalized with 50 peptides, which was higher than experiments (≈9), to ensure that the TmEnc protomer encounters them during a simulation, otherwise the AuNP‐protomer interaction effectively reduced to the case where AuNP was grafted only with ligands. In the presence of CLPs, a dense coverage of the AuNP surface was achieved using a ligand surface density of 4.46 ${\mathrm{nm}}^{-2}$ (350 ligands). In the absence of CLPs, a slightly higher density of 5.10 ${\mathrm{nm}}^{-2}$ (400 ligands) was used to ensure that the AuNP surface was sufficiently coated with ligands. These values fall within the range reported experimentally via NMR, where MUTAB surface density on AuNPs depends on particle size, increasing from 2.7 ± 0.7 ${\mathrm{nm}}^{-2}$ for 12.8 ± 1.6 nm particles to 5.5 ± 1.5 ${\mathrm{nm}}^{-2}$ for 4.5 ± 0.8 nm particles [69].

The overall model system, assembled by the PACKMOL software package [70], was electroneutral and had over 230,000 atoms. All simulations were performed using the GROMACS software package (version 2022.1) [71]. The spherical AuNP‐protomer PMF was computed via a series of over 30 umbrella sampling simulations in a cubic box of edge length ≈24 nm (fluctuating slightly under the NPT ensemble) containing a single AuNP and a single TmEnc protomer separated by a COM distance 5≲ζ≲10 nm with an interval of ≈0.15 nm. Similar protocol was used to evaluate the protomer‐protomer PMFs. Details of other simulation and analysis methods are described in Supporting Information.

## Conflicts of Interest

The authors declare no conflicts of interest.

## Supporting information


**Supporting File**: smll73690‐sup‐0001‐SuppMat.pdf.

## Data Availability

The data that support the findings of this study are openly available in ZFDM Repository UHH at https://www.fdr.uni‐hamburg.de/ , reference number 10.25592/uh‐hfdm.18192.
