# Supplementary material for: Peptide‐Ligand Cooperative Interplay Drives Gold Nanoparticle Encapsulation by Protein Cages
Source: Small. 2026 May 7;22(43):e73690. doi: 10.1002/smll.73690 (PMC13432550; doi:10.1002/smll.73690)
Supplement: Supplementary file 1 — Supporting File: smll73690‐sup‐0001‐SuppMat.pdf. [file SMLL-22-e73690-s001.pdf]

Supporting Information for Peptide-Ligand Cooperative Interplay Drives Gold Nanoparticle Encapsulation by Protein Cages

Wenhui Li   Niklas Mucke   Michael Rütten   Tommaso L. Schweers   Tobias Beck   Vikram Jadhao**

W.L. and N.M. Contributed equally to this work

Dr. Wenhui Li, Prof. Vikram Jadhao
Intelligent Systems Engineering, Indiana University Bloomington, Indiana 47408, USA
Email Address: vjadhao@iu.edu
Niklas Mucke, Dr. Michael Rütten, Tommaso L. Schweers
Department of Chemistry, Institute of Physical Chemistry, University of Hamburg, Hamburg 20146, Germany
Prof. Tobias Beck
Department of Chemistry, Institute of Physical Chemistry, University of Hamburg, Hamburg 20146, Germany
Hamburg Centre for Ultrafast Imaging, University of Hamburg, Luruper Chaussee 149, Hamburg, Germany
Email Address: tobias.beck@uni-hamburg.de

Experimental Methods

General Procedures

All syntheses and buffer preparations were performed using ultrapure water (18.2 MΩ·cm, Purelab Flex 2, ELGA LabWater). Glassware and magnetic stir bars used for gold nanoparticle synthesis were cleaned with aqua regia (3:1 HCl:HNO_3) and rinsed extensively with ultrapure water. Buffers were prepared from analytical grade reagents, filtered through 0.22 µm membrane filters, and degassed prior to chromatographic applications. Samples were handled in standard reaction vessels and stored at 4 °C unless stated otherwise. Centrifugation and sample concentration steps were conducted using standard benchtop centrifuges and centrifugal filters with appropriate molecular weight cut-offs (Amicon Ultra-15 Centrifugal Filters MWCO 30 or 100 kDa). Protein concentrations were determined with a NanoDrop One C spectrophotometer (Thermo Scientific) by measuring the absorbance at 280 nm and applying the Lambert–Beer law using the protein-specific extinction coefficient of $\varepsilon = 3.094 \times 10^4\,M^{-1}\,cm^{-1}$.

Chemicals

Hydrogen tetrachloroaurate trihydrate ($HAuCl_4 \cdot 3H_2O$), (11-mercaptoundecyl-)-*N,N,N*-trimethylammonium bromide (MUTAB), 11-mercaptoundecanoic acid (MUA), ethylenediaminetetraacetic acid (EDTA), Tris(hydroxymethyl) aminomethane (Tris), magnesium chloride hexahydrate ($MgCl_2 \cdot 6H_2O$), calcium chloride dihydrate ($CaCl_2 \cdot 2H_2O$), ammonium sulfate ($(NH_4)_2SO_4$), Cetyltrimethylammonium bromide (CTAB), Cetyltrimethylammonium chloride (CTAC) were obtained from Sigma-Aldrich. Trisodium citrate dihydrate ($Na_3C_6H_5O_7 \cdot 2H_2O$), dipotassium hydrogen phosphate trihydrate ($K_2HPO_4 \cdot 3H_2O$), isopropyl β-D-1-thiogalactopyranoside (IPTG), sodium chloride (NaCl), and SOB/TB media were purchased from Carl Roth. Citric acid ($C_6H_8O_7$) was obtained from Grüssing, glycerol from VWR, and uranyl acetate from Science Services. DNase I and RNase A were obtained from AppliChem. The cargo-loading peptide (CLP) was custom-synthesized by BioServUK, freeze-dried, and aliquoted into 1 mg batches for storage and use. All chemicals were used as received unless otherwise noted.

Inverse Turkevich Synthesis for MUTAB Stabilized Nanoparticles

Gold nanoparticles (AuNPs) were synthesized using an inverse Turkevich method, adapted from the protocol by Schulz et al. [1] with minor modifications. A typical synthesis for obtaining 12–13 nm particles was performed as follows: In an Erlenmeyer flask, trisodium citrate dihydrate (121.0 mg) and citric acid (29.0 mg) were dissolved in 200 mL ultrapure water. The solution was stirred (3 cm stir bar, 300 rpm) and heated to boiling, covered with a beaker to prevent solvent evaporation. Tetrachloroauric(III) acid trihydrate (16.0 mg) was dissolved in 5 mL ultrapure water and preheated to 80 °C. After boiling the citrate buffer for 14 minutes, EDTA (1.5 mg in 0.5 mL ultrapure water) was added. After one additional minute, the preheated gold precursor was rapidly added. A color change from colorless to wine-red indicated the formation of AuNPs. The reaction mixture was kept at boiling for another 20 minutes, then cooled to room temperature and stored at 4 °C until further use.

Seed-Mediated Growth of MUA Stabilized Gold Nanoparticles

MUA-stabilized gold nanoparticles were synthesized via a seed-mediated growth protocol adapted from Schulz et al. [1] and Zheng et al. [2] with minor modifications.
Seed synthesis: An aqueous CTAB solution (9.9 mL, 100 mM) was mixed with $HAuCl_4$ (100 µL, 25 mM) at 27 °C in a thermostated water bath and stirred at 600 rpm. Freshly prepared $NaBH_4$ (600 µL, 10 mM) was rapidly injected into the mixture. After stirring for 3 min, the reaction was left undisturbed at 27 °C for 3 h. The resulting gold seed solution was used without further purification.
Growth step: For the growth of 12 nm particles, 200 µL of the seed solution was added to a growth mixture containing ascorbic acid (15 mL, 100 mM) and CTAC (20 mL, 200 mM) under stirring (600 rpm,

27 °C). A $HAuCl_4$ solution (20 mL, 0.5 mM) was then added dropwise while stirring at 300 rpm and 27 °C. The reaction was maintained under these conditions for 15 min to complete growth.
Purification: To remove excess CTAC and byproducts, the AuNP dispersion was extracted with dichloromethane (DCM) and centrifuged at 3,220 × g for 10 min at 20 °C. The aqueous phase containing the nanoparticles was collected and concentrated using centrifugal filter units (MWCO 30 kDa). Further purification was performed by two sequential sedimentation/redispersion cycles (20,000 × g, 20 min, 20 °C) in ultrapure water. The final particle dispersion was stored at room temperature until use.

Ligand Exchange with MUTAB

To render the citrate-stabilized AuNPs positively charged, a ligand exchange was performed using 11-(mercaptoundecyl)-N,N,N-trimethylammonium bromide (MUTAB). A 100-fold molar excess of MUTAB (46 mg), calculated relative to the estimated number of surface ligands per nanoparticle, was dissolved in 10 mL of 2 M HCl. This solution was added to 200 mL of AuNP dispersion, resulting in a final HCl concentration of 0.1 M. The mixture was incubated at room temperature for 48 h without agitation. Excess ligand was removed by centrifugal filtration (MWCO 30 kDa). The AuNPs were washed five times with 0.1 M HCl and five times with ultrapure water, then concentrated to 1 mL and stored at 4 °C.

Surface Functionalization with Cargo Loading Peptide (CLP)

For peptide attachment, a CLP stock solution (1.0 mg/mL in water-free DMF) was prepared. MUTAB-stabilized AuNPs were mixed with an amount of CLP corresponding to 9 or 50 peptides per nanoparticle. The suspension was diluted 1:10 with DMF and incubated for 16 h at room temperature. Subsequently, the mixture was diluted 1:10 with water, followed by purification and concentration using centrifugal filters (MWCO 30 kDa), washed five times with ultrapure water, and stored at 4 °C until use.

Ligand Exchange with MUA

For the ligand exchange on 13 nm gold nanoparticles synthesized via seed-mediated growth, freshly prepared sodium borohydride ($NaBH_4$; 350 µL, 50 mM) was added to 500 µL of purified AuNP solution. The mixture was incubated in a thermomixer (20 °C, 1000 rpm, 1 h). Subsequently, a solution of 11-mercaptoundecanoic acid (MUA; 350 µL, 50 mM in 50% v/v ethanol) was added, and the sample was incubated for an additional 1 h under the same conditions. Afterward, the reaction mixture was left undisturbed at room temperature overnight to complete ligand exchange.

Preparation of Reassembled Encapsulin under Different Ionic Strength Conditions

Encapsulin was disassembled by acidification: 1.0 mg of Encapsulin (50 µL of a 20 mg/mL stock in 20 mM Tris-HCl, pH 7.5, 0.3 M NaCl) was diluted tenfold with 500 µL of 10 mM phosphate buffer (pH 1.0) and incubated at 4 °C for 1 h. Reassembly was initiated by a 1:100 dilution into 50 mL of 20 mM phosphate buffer (pH 7.0), followed by overnight incubation at room temperature. The reassembled protein was concentrated using centrifugal filters (MWCO 30 kDa) and stored at 4 °C until further processing (e.g., FPLC or TEM). To test the effect of ionic strength on reassembly, reassembly buffers were supplemented with 5 M NaCl to final concentrations of 50 mM, 750 mM, or 1.5 M.

Protein Production

Protein production includes molecular cloning and protein expression and purification.
Molecular Cloning: Chemically competent *E. coli* C43 (DE3) cells (100 µL) were thawed on ice for 10 min and transformed with 200 ng of plasmid DNA (pET-22b(+), GenScript). The mixture was incubated on ice for 30 min, heat-shocked at 42 °C for 45 s, and placed on ice for another 2 min. Cells were recovered in 0.9 mL SOB medium at 37 °C for 1 h with gentle agitation.

Cells were pelleted (1,000 × g, 1 min), resuspended in 100 µL SOB medium, plated on LB-agar supplemented with ampicillin (150 µg/mL), and incubated overnight at 37 °C. A single colony was used to inoculate 5 mL LB medium with ampicillin (150 µg/mL), incubated overnight (37 °C, 250 rpm), mixed 1:1 with 50% (w/w) glycerol, and stored at -80 °C.

Protein Expression and Purification: Cryostocks were used to inoculate 4 mL of Terrific Broth (TB) containing ampicillin (150 µg/mL), incubated overnight (37 °C, 250 rpm). The preculture (4 mL) was added to 400 mL sterile TB medium with ampicillin (150 µg/mL) and cultivated at 37 °C until an OD_{600} of 0.7 was reached. Protein expression was induced with IPTG (0.5 mM final concentration) and cultures were harvested after 4 h by centrifugation (4,000 × g, 10 min, RT).

Cell pellets were resuspended in 20 mL lysis buffer (50 mM Tris-HCl, pH 9.0) supplemented with RNase A and DNase I. Cells were lysed by sonication (Vibra-Cell VCX-130; 15 cycles, 60% amplitude, 59 s on/59 s off on ice). Lysates were cleared by centrifugation (14,000 × g, 20 min, 4 °C), and the supernatant was supplemented with $MgCl_2$ (2.5 mM) and $CaCl_2$ (0.5 mM), followed by incubation at 37 °C for 4 h to promote nuclease activity.

Insoluble material was removed (14,000 × g, 15 min, 4 °C), and the clarified supernatant was heat-treated (65 °C, 10 min), followed by a second centrifugation (14,000 × g, 15 min, 4 °C). Proteins were precipitated using ammonium sulfate (70% saturation), pelleted (14,000 × g, 25 min, 4 °C), redissolved in 20 mL Tris-HCl buffer (50 mM, pH 9.0), and filtered (0.22 µm).

Protein purification was carried out by anion-exchange chromatography (ÄKTA go FPLC system, Cytiva) on a 5 mL HiTrap^{TM} Q HP column (Cytiva). Proteins were eluted with a linear NaCl gradient (0–1 M). Fractions eluting at 46 mS/cm conductivity were pooled and concentrated.

Final purification was performed via size exclusion chromatography (SEC) on a Superose 6 Increase 10/300 GL column (Cytiva) using SEC buffer (20 mM Tris-HCl, pH 7.5; 0.3 M NaCl). Fractions eluting at 12.7 mL were pooled. The purified protein was stored in SEC buffer at 4 °C until use.

Nanoparticle Characterization

The synthesized AuNPs were characterized to confirm their size, surface charge, morphology, and ligand coverage. Dynamic light scattering (DLS) and ζ-potential measurements were used to determine the hydrodynamic diameter and surface charge, respectively. Thermogravimetric analysis (TGA) was employed to estimate the ligand coverage on the nanoparticle surface. Temperature-dependent UV–Vis spectroscopy was employed to evaluate thermal stability.

Dynamic Light Scattering and ζ-Potential Measurements

DLS and ζ-potential measurements were performed using a Zetasizer Pro Blue (Malvern Panalytical) equipped with a He–Ne laser ($\lambda = 633$ nm) and a backscattering angle of 173°. Samples were equilibrated to 25 °C prior to analysis and measured in DTS1070 disposable folded-capillary cells (Malvern Panalytical). Data evaluation was carried out using the ZS Xplorer software.

For DLS, the hydrodynamic radius was obtained from three consecutive runs and reported as the software-averaged value (Fig. S7(a)). For ζ-potential measurements, each value represents the average of three runs (Fig. S7(b)).

Thermogravimetric Analysis

TGA measurements were performed on a TGA 209 F1 Libra instrument (NETZSCH-Gerätebau GmbH). MUTAB-functionalized Au nanoparticles were dried at 65 °C prior to analysis and transferred into standard alumina crucibles. Measurements were conducted under a constant N_2 flow (20 mL/min), applying a heating rate of 10 K min^{-1} from 25 to 500 °C. The instrument was baseline-corrected using an empty crucible under identical conditions. Results are shown in Fig. S8.

Temperature-dependent UV–Vis spectroscopy

Absorbance spectra were recorded on a Cary 60 UV–Vis spectrophotometer (Agilent Technologies) equipped with a Single Cell Peltier temperature controller (SPV 1x0). Quartz cuvettes (Hellma Analytics) with a 2 mm path length were used. Ultrapure water was used as the reference. Measurements were conducted over a temperature range of 25 to 70 °C. Results are shown in Fig. S9.

Simulation Methods

Computational Model

The TmEnc protomer, MUTAB and MUA ligands, CLP, AuNP, NaCl salt ions, and water molecules were modeled using the Martini force field (version 3.0) [3]. Martini is a widely used coarse-grained force field in biomolecular simulations [3], mapping 2, 3, or 4 heavy atoms into one bead of tiny, small, or regular size respectively. Beads are categorized based on polarity: P (polar), N (non-polar), and C (apolar). Additional categories include X (halo-compounds), Q (monovalent charged), D (divalent charged), and W (water). Each bead category includes various subtypes to further specify polarity and additional properties. Interactions between beads are classified into two types: bonded and non-bonded. Bonded interactions consist of bond stretching and angle bending, both described by harmonic potentials. Non-bonded interactions between beads include pairwise van der Waals interactions, represented by a 12-6 Lennard-Jones potential, and electrostatic (Coulomb) interactions. The Martini coarse-graining imparts each TmEnc protomer, ligand, and CLP with a net charge of $-13e$, $+1e$, and 0 respectively. Coulomb interactions in Martini 3.0 force field are evaluated with non-polarizable water model using the reaction field method [4] with a relative dielectric constant of 15 within the cutoff distance (1.1 nm), transitioning to an effectively infinite dielectric constant beyond the cutoff. A correction term ensures that the electrostatic interaction at the cutoff distance is zero. Results obtained using this non-polarizable model were found to be qualitatively similar to the results obtained using the Martini 2.2 force field which employs a polarizable water model (see Fig. S6), where Coulomb interactions are processed using the particle-mesh Ewald algorithm [5] with a relative dielectric constant of 2.5.
The coarse-grained model structures for the TmEnc protomer, MUTAB ligand, CLP, and bare AuNP core are shown in Fig. S1. The force field parameters for protomer, ligand, and CLP were assigned using the python script *Martinize2* [6] that converts the all-atom topology to the Martini coarse-grained version. The AuNP core was modeled as a solid sphere of diameter $\approx$ 5 nm comprising 3,925 gold atoms, which translate into 3,925 regular-sized apolar (C6) beads following a 1:1 coarse-grained mapping scheme. The model AuNP size was chosen to be smaller than that used in experiments (diameter $\approx$ 13 nm) due to computational limitations. The smaller AuNP size and increased curvature may influence the contact area between the TmEnc protomer and the AuNP, thereby affecting their interaction. These size effects were probed by computing the protomer-AuNP interaction for an AuNP modeled as a planar wall (see Fig. S2). A water molecule was modeled as a single tiny-sized bead, which produced a protomer-protomer interaction independent of salt concentration (see Fig. S4), consistent with experiments.

Simulation Details

All simulation systems were assembled by the PACKMOL software package [7] and performed using the GROMACS software package (version 2022.1) [8, 9]. First, the energy of the system was minimized by the steepest descent algorithm to ensure that the maximum force on any bead is smaller than 500 kJ/(mol·nm). For systems containing the spherical AuNP, a 10-ns molecular dynamics (MD) simulation in NPT ensemble was subsequently performed at the specified temperature (277 K, 300 K, or 310 K) and ambient pressure (1 bar) to relax the solvent, keeping the positions of the center atoms of AuNP and TmEnc protomer fixed in order to maintain their original center of mass (COM) distance. For systems containing a gold plane (see Fig. S2), a 10-ns MD simulation in NVT ensemble was performed to

relax the solvent, with the gold plane kept frozen and the position of the center atom of the protomer restrained. Afterward, a steered MD simulation was conducted to move the protomer toward the AuNP (or gold plane) at a rate of 0.005 nm/ps. This step generated a series of configurations along the reaction coordinate (ζ), which were used as initial configurations for the subsequent umbrella sampling simulations. The distance interval between two neighboring sampling windows was set to ≈ 0.15 nm. Finally, 200-ns umbrella sampling simulations with a time step of 10 fs were performed, and the trajectory data from the last 150 ns was used for analysis. Longer simulations (e.g., 350 ns with the last 300 ns used for analysis) showed negligible change in the potential of mean force (PMF) curves. During the umbrella sampling, a harmonic position restraint with a force constant of 1000 kJ/(mol·nm^2) was applied to constrain the relative distance between AuNP (or gold plane) and protomer. For the systems containing the 5-nm AuNP, this distance refers to the three-dimensional Euclidean distance between the COMs of AuNP and protomer. For the systems containing a gold plane, it corresponds to the distance in the z direction between the COMs of the gold plane and the protomer (see Fig. S2 caption). The COM position and force on the protomer were recorded every 500 fs. After the umbrella sampling simulations, the Weighted Histogram Analysis Method (WHAM) [10, 11] was used to calculate the PMF.

A series of independent simulation systems containing only a 5-nm AuNP immersed in water with varying salt concentrations (0, 200, 500, and 800 mM) was set up to assess the effects of ligands on peptide conformation, measured via the angle ϕ (see Fig. 4(b) in the main article). Two scenarios were considered: AuNP functionalized with 350 ligands and 50 peptides, and AuNP functionalized with only 50 peptides. After energy minimization and a 20-ns MD simulation in NPT ensemble for equilibration, a 200-ns NPT simulation with a 10-fs time step was performed for sampling the equilibrium structure of the functionalized AuNP, and the data from the final 150 ns was used for analysis. These simulations were performed in a cubic box of edge length ~ 20 nm, fluctuating slightly under NPT conditions. The configurations during sampling were saved every 0.1 ns.

In all simulations, temperature was maintained via a thermostat based on the v-rescale algorithm [12] with a coupling time of 1 ps, and the pressure was controlled via a barostat based on the c-rescale algorithm [13] with a time constant of 4 ps. A leap-frog algorithm [14] was used to integrate Newton's equations of motion. Three-dimensional periodic boundary conditions (PBCs) were applied. Lennard-Jones potential was truncated and shifted to 0 at 1.1 nm.

Peptide Binding Probability

The probability of a CLP to bind the TmEnc protomer surface shown in Fig. 3(e) in the main article was computed using the following method. CLPs whose COM of the anchor sequence (see Fig. 3(f) inset in the main article) lie within 1.75 nm of the protomer's COM were classified as interacting with the protomer. For each interacting CLP, the distance between each bead of CLP's anchor sequence and all protomer beads was computed. Binding sites on the protomer surface were identified as protomer beads that lie within 0.5 nm of any bead of the CLP anchor sequence. The binding probability of each protomer bead was computed based on its frequency of interaction with the CLPs evaluated using the trajectory frames. These frames were selected based on the condition that the AuNP-protomer COM distance is within the range of 5.0 to 5.5 nm, where the AuNP-protomer free energy is near its minimum. The choice of 1.75 nm and 0.5 nm for the classification and binding criteria respectively was guided by geometric considerations. Given the plate-like geometry of the protomer, we define its end-to-end thickness as the distance measured along the direction normal to the major surface, representing the minor structural dimension of the protomer. The TmEnc protomer has a thickness of ≈ 2.5 nm, and a typical bead has a diameter of ≈ 0.5 nm. When a CLP is in contact with the surface of the protomer, the minimum distance between their COMs is 1.5 nm. Allowing for a 0.25 nm buffer, 1.75 nm was chosen for the classification criterion. Similarly, if a protomer bead is in contact with any bead in the CLP anchor sequence, their separation is ≈ 0.5 nm.

Peptide Flexibility

The peptide flexibility is assessed via the angle θ (see Fig. 3(f) inset in the main article) that measures the CLP's response to incoming protomers as a function of the distance d between the backbone bead of the end amino acid in a CLP and the protomer COM. For a given salt concentration, $\theta(d)$ was extracted using all the reaction coordinates shown in Fig. 2(d) of the main article at the same salt concentration. Each reaction coordinate is associated with 150 trajectory frames (produced via the last 150 ns simulation), and in each frame we have 50 CLPs producing a $\theta(d)$ sample. Using the aggregate dataset combined over all CLPs and frames, the distances d were binned with a window (bin width) of 0.46 nm, and the corresponding average θ with the standard error was computed. A range of bin sizes from 0.1 nm to 1.0 nm were considered and a bin size of 0.46 nm was selected based on the trade-off between resolution and statistical reliability: bin sizes that were too small lacked sufficient data points to yield statistically meaningful averages, while larger bins tended to smooth out important features in the data. The plotted distance range in Fig. 3(f) of the main article spans from 1.5 nm to 7.0 nm. Insignificant variations in θ were noted beyond 7.0 nm, and too few data points were obtained below 1.5 nm (which is approximately the contact distance) to yield meaningful statistics.

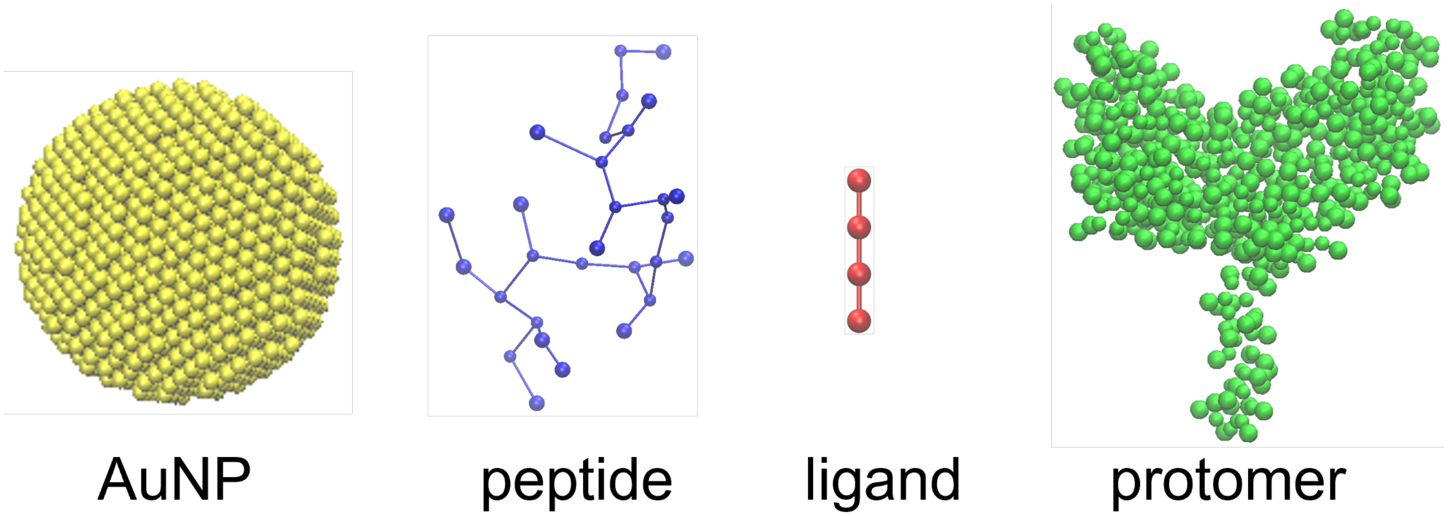

Figure S1: From left to right: Coarse-grained Martini model structures of a bare gold nanoparticle (AuNP), peptide, ligand, and protomer. The bare AuNP is represented as a solid sphere with a diameter of ≈ 5 nm, composed of 3,925 beads. The peptide consists of 16 amino acids and is modeled with 29 beads. The ligand is a linear molecule represented by 4 beads. The protomer is modeled using 616 beads.

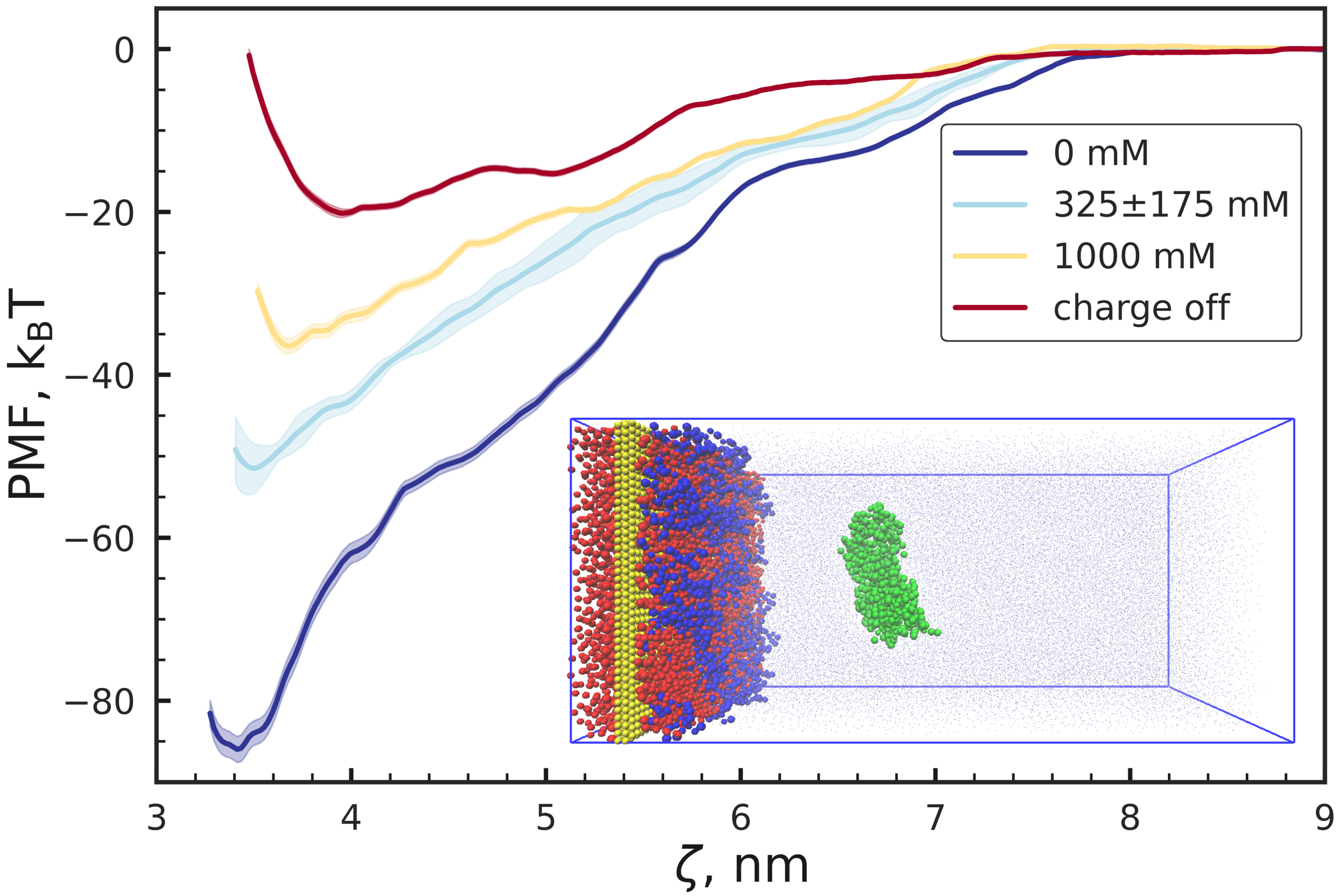

Figure S2: To investigate the effect of gold nanoparticle (AuNP) size on the potential of mean force (PMF), a gold plane is grafted with ligands and peptides using the same surface density as in Fig. 2(d) of the main article. The gold plane matches the x-y plane dimensions of the simulation box (12.23×12.11 nm^2) and has a thickness of four layers of gold atoms (~ 0.6 nm). A protomer centered in the x-y plane is positioned ≈ 9 nm above this plane along the z direction. In this setup, the periodic boundary conditions (PBCs) render the gold plane effectively infinite, simulating an AuNP with zero curvature. The resulting PMFs at different NaCl concentrations (legend) reveal deeper minimum values compared to Fig. 2(d) in the main article, attributed to the increased contact area between the protomer and the gold plane. The difference diminishes with increasing salt concentration. The "charge off" case produces a lower bound of the free-energy minimum (-20 $k_B T$) for the co-assembly product of AuNPs outside empty encapsulin cages (Fig. 2(d)). Similarly, the PMF for 325 ± 175 mM produces a lower bound of the free-energy minimum (-50 $k_B T$) for the case of successful encapsulation. The bands with corresponding lighter colors represent the standard errors. ζ represents the center of mass distance between the protomer and the gold plane in the z direction.

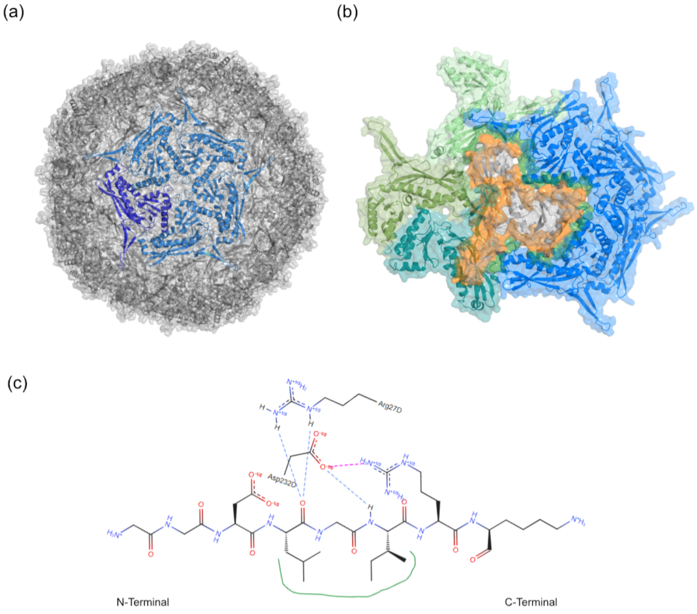

Figure S3: Structural organization of the Encapsulin cage and CLP binding interface. (a) Surface representation of the Encapsulin nanocage highlighting one pentameric unit (blue) and a single subunit within this pentamer (purple). (b) Single Encapsulin subunit with all subunit–subunit interfaces visualized; the orange regions indicate the interface areas between neighboring subunits, shown in different colors (green, blue, and cyan) to illustrate their spatial arrangement. (c) Binding mode of the CLP binding motif within the Encapsulin binding pocket. Dashed lines depict hydrogen-bond interactions, and the green contour marks the hydrophobic interaction region. Only the truncated CLP segment present in the crystal structure (PDB: 3DKT) is shown. The CLP interacts primarily through its Leu backbone with Arg27 and through its Arg residue with Asp232 via hydrogen bonding and electrostatic interactions. Additionally, Ile and Leu residues form hydrophobic contacts within the nonpolar region of the CLP binding pocket. The interaction diagram was generated using PoseEdit on the ProteinsPlus server (University of Hamburg) [15].

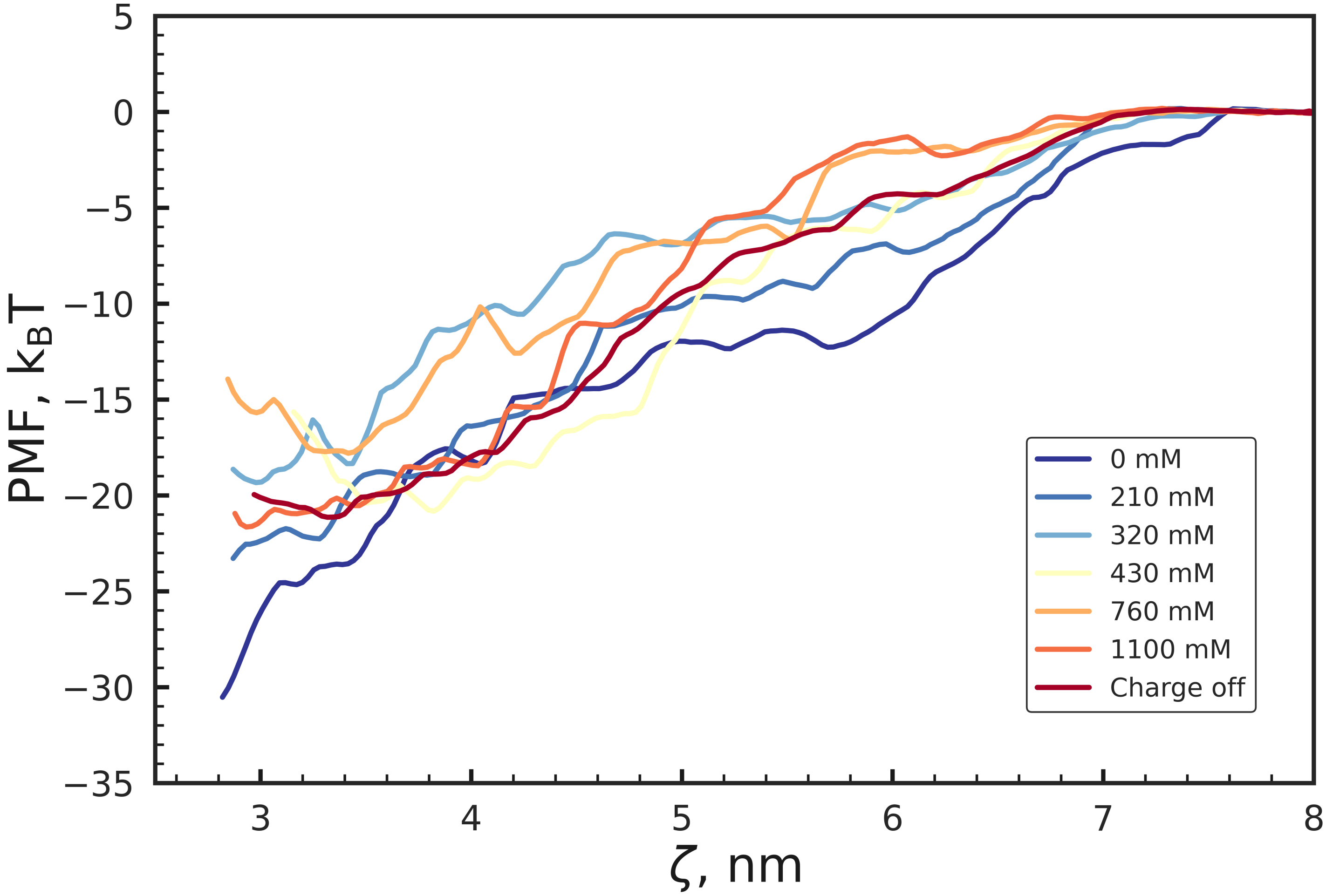

Figure S4: Effect of NaCl concentration on the potential of mean force (PMF) associated with the interaction between two protomers solvated in water modeled using a Martini tiny-sized bead representation. The reaction coordinate ζ represents the center of mass (COM) distance between the two protomers. The interaction between protomers is largely unaffected by changes in salt concentration. The "charge off" case represents highly screened electrostatic interactions.

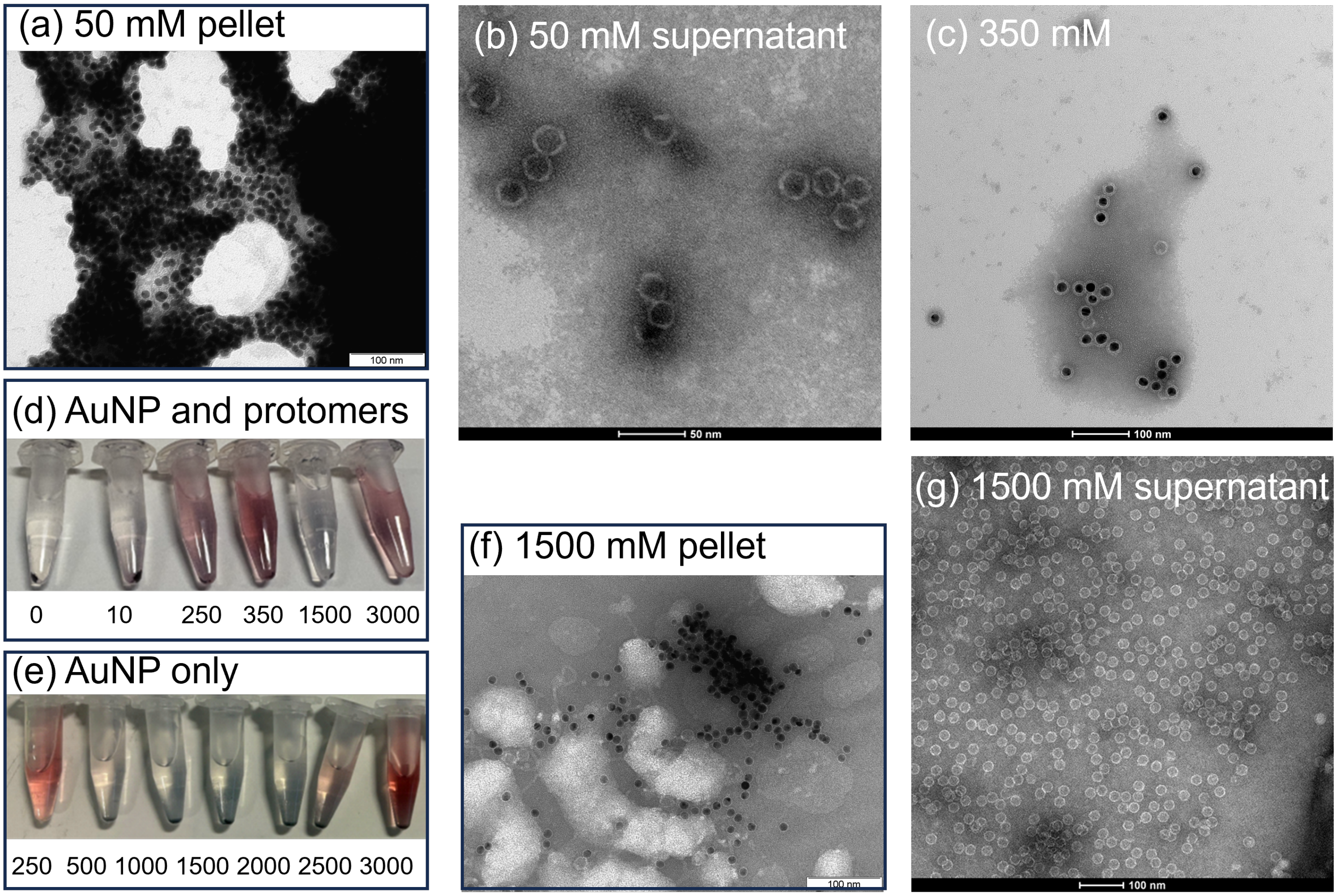

Figure S5: The AuNPs in this figure are functionalized with MUTAB ligands and CLPs. (a) At 50 mM salt, the pellet forms an amorphous protein–AuNP matrix lacking defined structure, which are identified as AuNP–protomer co-precipitates. Due to this co-precipitation, a considerable fraction of protein is unavailable for productive cage self-assembly. (b) At 50 mM salt, the supernatant only contains empty protein cages. (c) At 350 mM salt, a representative TEM image demonstrates successful encapsulation, with an encapsulation efficiency of 55.6%. (d) Photographs of encapsulation reactions after incubation and centrifugation in various salt concentrations (unit: mM) denoted in the bottom. Stable AuNPs and homogeneous color distribution are observed at moderate ionic strengths (250 and 350 mM), while at a higher salt concentration (1500 mM) only precipitated AuNPs occur. At 3000 mM salt concentration, AuNPs appear stable again, as indicated by a uniform color distribution and minimal sedimentation. (e) Photographs of AuNPs in various salt concentrations (unit: mM) denoted in the bottom. Only AuNPs in the solutions in the range of 500 to 2500 mM salt are not stable. (f) At 1500 mM salt, the pellet shows that AuNPs are unstable and precipitate. (g) At 1500 mM salt, protomers form empty encapsulin cages only; their abundance is higher than at lower salt concentrations (e.g., 50 mM).

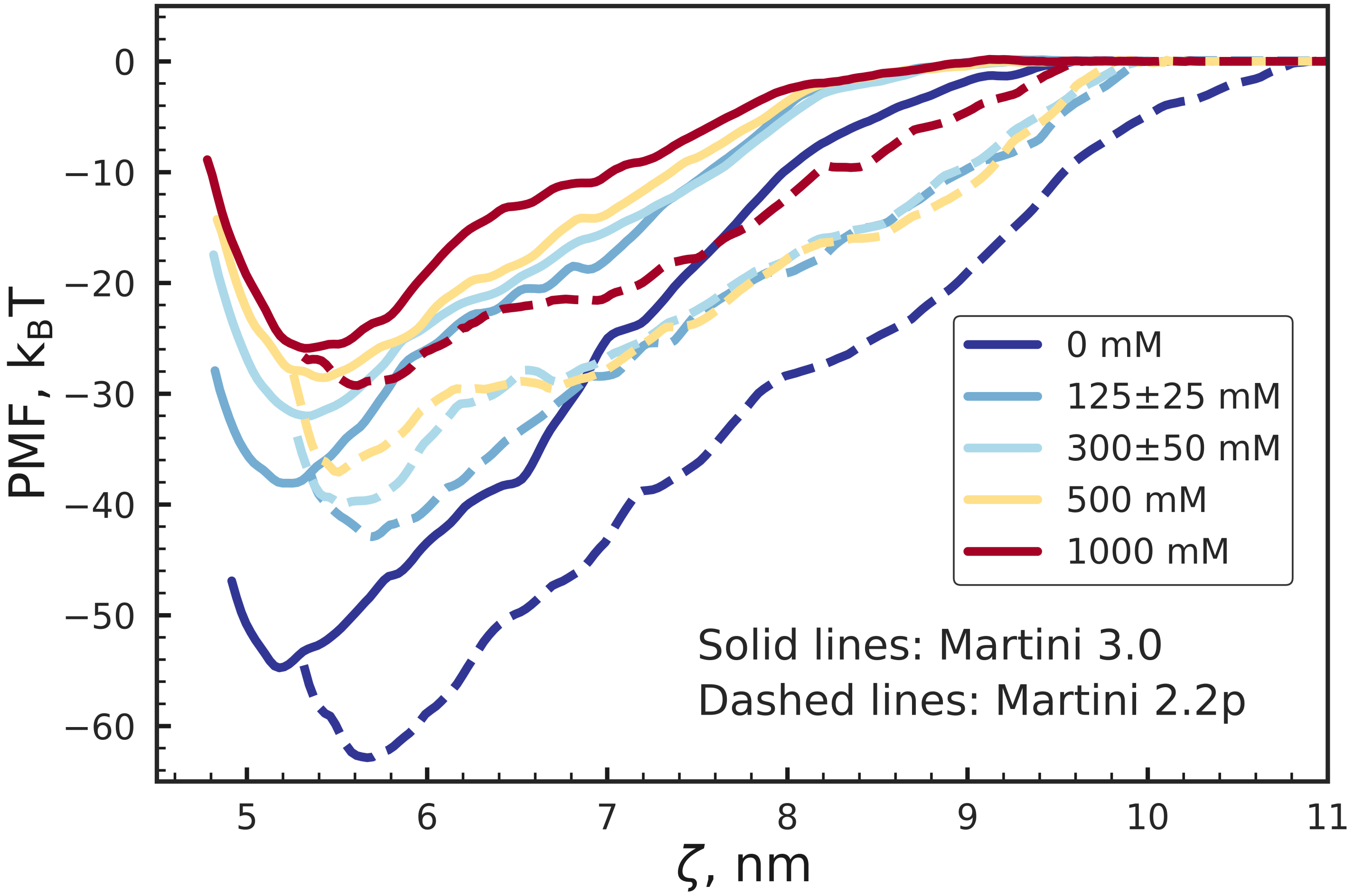

Figure S6: Comparison of AuNP-protomer PMFs using Martini 3.0 force field with non-polarizable water model and Martini 2.2p force field with polarizable water model. The AuNP is functionalized by ligands and peptides as in Fig. 2(d) of the main article. In the non-polarizable model, water is represented by a single bead without partial charges. In the polarizable model, each water bead is connected to two dummy particles carrying opposite charges [16]. This approach allows the explicit consideration of electrostatic interactions involving water. However, the force field parameters for other components must be reverted to those from the earlier Martini version 2.2p [17, 16], as the current Martini 3.0 is not compatible with polarizable water. While the overall trends as a function of NaCl concentration are qualitatively the same across the two models, we do observe a few quantitative differences. AuNP-protomer PMFs obtained using Martini 2.2p model exhibit a longer interaction range (∼ 1 nm longer decaying to zero) and larger contact distance (∼ 0.5 nm larger) as well as higher binding free energy (∼ $5 - 10$ $k_B T$) compared to the Martini 3.0 results. The longer interaction range and stronger attraction observed with Martini 2.2p can be attributed to a different treatment of electrostatic interactions and a different set of force-field parameters. Martini 2.2p employs particle-mesh Ewald (PME) summation [5], whereas Martini 3.0 uses a reaction field approach [4] for long-range interactions. On the other hand, Martini 3.0 is a finer coarse-grained model, with a more refined and optimized set of force-field parameters, compared to Martini 2.2p. For example, Martini 2.2p uses a generic bead of diameter 0.47 nm to represent water while Martini 3.0 has a specific bead type for water (here, the tiny-sized bead of diameter 0.34 nm was used). The larger contact distance observed with Martini 2.2p can be attributed to its coarser bead resolution.

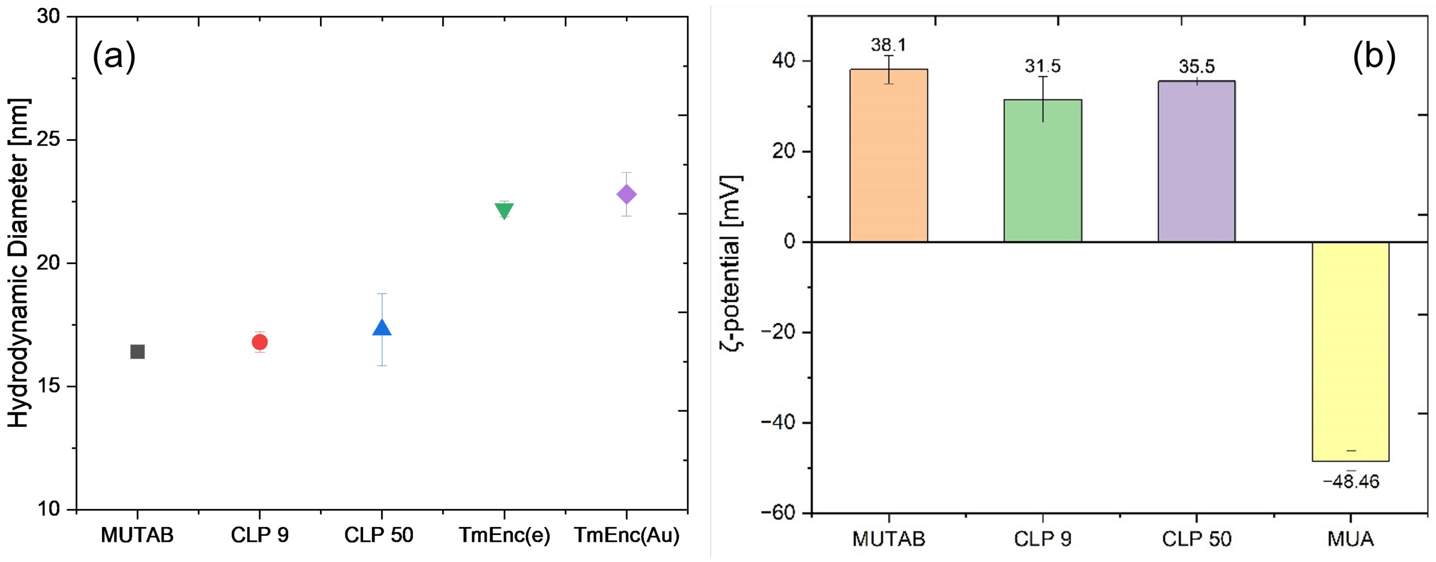

Figure S7: (a) Volume-weighted hydrodynamic diameters of differently functionalized AuNPs and the encapsulin–AuNP complexes determined by DLS. AuNPs capped only with MUTAB show an average diameter of 16.4 nm, while the addition of 9 and 50 CLP peptides increases the size slightly to 16.8 nm and 17.7 nm, respectively. The native encapsulin cage shows a size of 22.2 nm, and the encapsulated AuNP shows a size of 22.8 nm. The slightly smaller DLS-derived hydrodynamic diameter (22 nm vs. 24 nm from crystallography) reflects the difference between diffusion-based and geometric size measurements. Error bars represent the standard deviation from three independent measurements. (b) ζ-potentials of AuNPs functionalized with different ligands. MUTAB−, CLP(9)−, and CLP(50)−AuNPs show positive surface charges (38.1, 31.5, and 35.5 mV), while MUA−AuNPs exhibit a negative ζ-potential (−48.5 mV).

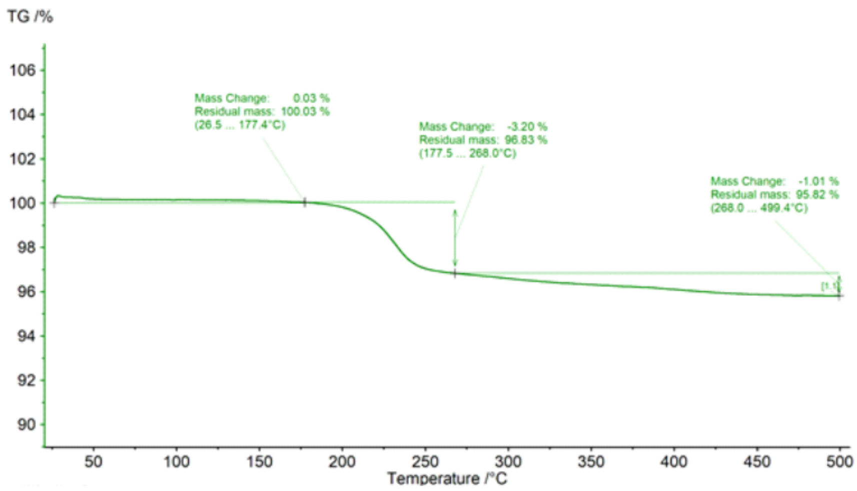

Figure S8: Thermogravimetric analysis (TGA) of MUTAB-functionalized AuNP. TGA recorded under N_2 atmosphere (10 K min^{-1}, 25–500 °C) shows a two-step mass loss: 3.2% between 177–268 °C and an additional 1.0% between 268–500 °C, corresponding to a total organic mass loss of 4.2%. The main step is assigned to thermal decomposition of the MUTAB ligand layer, while the minor high-temperature contribution arises from residual organics more strongly bound to the Au surface. Assuming solely MUTAB ($M = 326.38$ g mol^{-1}), the mass loss corresponds to approximately 2000 ligands per nanoparticle, or 3.6 ligands nm^{-2} for an average particle diameter of 13.3 nm. The obtained surface coverage is consistent with reported values for densely packed cationic surfactant layers on AuNP [18].

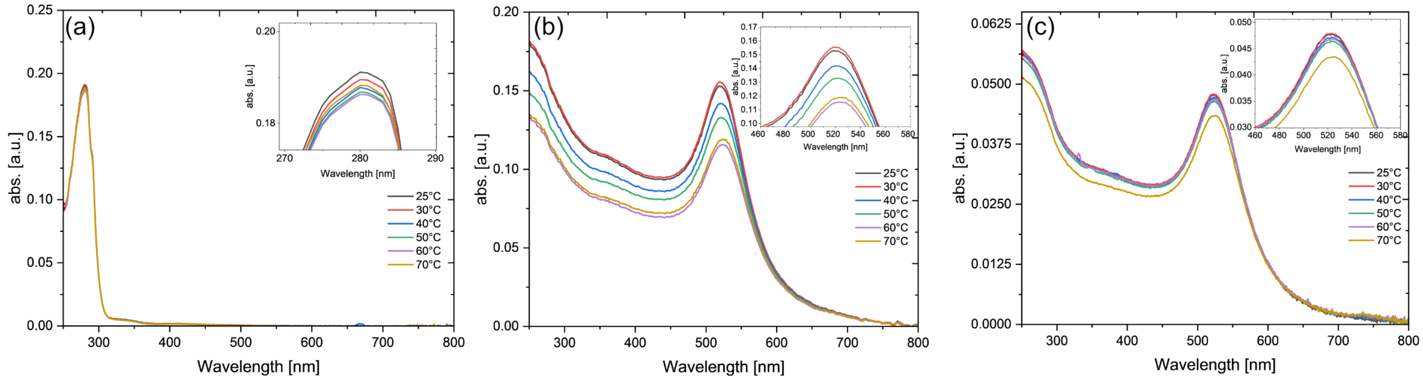

Figure S9: Temperature-dependent UV–Vis spectra of encapsulin cages, gold nanoparticle and encpasulated gold nanoparticles. (a) Empty encapsulin protein cages (Enc) showing a characteristic protein absorption band around 280 nm with minimal temperature dependence. (b) MUTAB- and CLP-functionalized gold nanoparticles (AuNPs) exhibiting a surface plasmon resonance (SPR) band at 520 nm with decreasing intensity and slight red shift to 524 nm SPR band at elevated temperatures. (c) Encapsulin loaded with gold nanoparticles (Enc(Au)), displaying dominantly the AuNP SPR band. Increasing temperature leads to a reduction in absorbance above 60°C and a slight redshift from 522 nm to 524 nm. Below 60°C the SPR peak position remains largely unchanged. Insets show magnified views of the respective spectral regions.

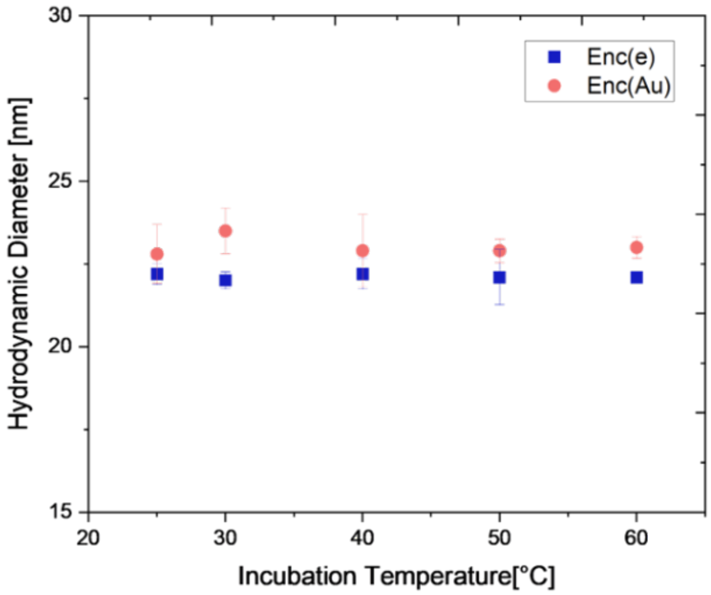

Figure S10: Temperature-dependent stability of encapsulin cages with and without encapsulated AuNPs. The hydrodynamic diameter of empty encapsulin (Enc(e), blue squares) and AuNP-loaded encapsulin (Enc(Au), red circles) was measured by DLS after 30 min incubation at the indicated temperatures. No significant change in hydrodynamic diameter was observed up to 60 °C, indicating that both the empty and AuNP-loaded protein cages remain structurally stable under these conditions. Error bars represent standard deviations from three independent measurements.

References

[1] F. Schulz, T. Homolka, N. G. Bastus, V. Puntes, H. Weller, T. Vossmeyer, *Langmuir* **2014**, *30*, 35 10779.

[2] Y. Zheng, X. Zhong, Z. Li, Y. Xia, *Particle & Particle Systems Characterization* **2014**, *31*, 2 266.

[3] P. C. Souza, R. Alessandri, J. Barnoud, S. Thallmair, I. Faustino, F. Grünewald, I. Patmanidis, H. Abdizadeh, B. M. Bruininks, T. A. Wassenaar, et al., *Nature methods* **2021**, *18*, 4 382.

[4] M. Neumann, *The Journal of chemical physics* **1985**, *82*, 12 5663.

[5] T. Darden, D. York, L. Pedersen, *The Journal of chemical physics* **1993**, *98*, 12 10089.

[6] P. C. Kroon, F. Grünewald, J. Barnoud, M. van Tilburg, P. C. Souza, T. A. Wassenaar, S.-J. Marrink, *arXiv preprint arXiv:2212.01191* **2022**.

[7] L. Martínez, R. Andrade, E. G. Birgin, J. M. Martínez, *Journal of computational chemistry* **2009**, *30*, 13 2157.

[8] D. Van Der Spoel, E. Lindahl, B. Hess, G. Groenhof, A. E. Mark, H. J. Berendsen, *Journal of computational chemistry* **2005**, *26*, 16 1701.

[9] M. J. Abraham, T. Murtola, R. Schulz, S. Páll, J. C. Smith, B. Hess, E. Lindahl, *SoftwareX* **2015**, *1* 19.

[10] S. Kumar, J. M. Rosenberg, D. Bouzida, R. H. Swendsen, P. A. Kollman, *Journal of computational chemistry* **1992**, *13*, 8 1011.

[11] A. Grossfield, Wham: the weighted histogram analysis method, **2012**.

[12] G. Bussi, D. Donadio, M. Parrinello, *The Journal of chemical physics* **2007**, *126*, 1.

[13] M. Bernetti, G. Bussi, *The Journal of Chemical Physics* **2020**, *153*, 11.

[14] W. F. Van Gunsteren, H. J. Berendsen, *Molecular Simulation* **1988**, *1*, 3 173.

[15] K. Diedrich, B. Krause, O. Berg, M. Rarey, *Journal of Computer-Aided Molecular Design* **2023**, *37*, 10 491.

[16] S. O. Yesylevskyy, L. V. Schäfer, D. Sengupta, S. J. Marrink, *PLoS computational biology* **2010**, *6*, 6 e1000810.

[17] D. H. De Jong, G. Singh, W. D. Bennett, C. Arnarez, T. A. Wassenaar, L. V. Schafer, X. Periole, D. P. Tieleman, S. J. Marrink, *Journal of chemical theory and computation* **2013**, *9*, 1 687.

[18] K. M. Hatzis, X. Wei, M. Kincanon, A. Wo, J. Gandrapu, O. Zeiri, R. Hernandez, C. J. Murphy, *Chemistry of Materials* **2025**.
